# Insight into Phosphatidylinositol-Dependent Membrane Localization of the Innate Immune Adaptor Protein Toll/Interleukin 1 Receptor Domain-Containing Adaptor Protein

**DOI:** 10.3389/fimmu.2018.00075

**Published:** 2018-01-29

**Authors:** Mahesh Chandra Patra, Sangdun Choi

**Affiliations:** ^1^Department of Molecular Science and Technology, Ajou University, Suwon, South Korea

**Keywords:** toll-like receptor, toll/interleukin 1 receptor domain-containing adaptor protein, phosphatidylinositol 4,5-bisphosphate, phosphatidylinositol (3,4,5)-trisphosphate, molecular dynamics simulation, molecular mechanics/Poisson–Boltzmann surface area, membrane localization

## Abstract

The toll/interleukin 1 receptor (TIR) domain-containing adaptor protein (TIRAP) plays an important role in the toll-like receptor (TLR) 2, TLR4, TLR7, and TLR9 signaling pathways. TIRAP anchors to phosphatidylinositol (PI) 4,5-bisphosphate (PIP2) on the plasma membrane and PI (3,4,5)-trisphosphate (PIP3) on the endosomal membrane and assists in recruitment of the myeloid differentiation primary response 88 protein to activated TLRs. To date, the structure and mechanism of TIRAP’s membrane association are only partially understood. Here, we modeled an all-residue TIRAP dimer using homology modeling, threading, and protein–protein docking strategies. Molecular dynamics simulations revealed that PIP2 creates a stable microdomain in a dipalmitoylphosphatidylcholine bilayer, providing TIRAP with its physiologically relevant orientation. Computed binding free energy values suggest that the affinity of PI-binding domain (PBD) for PIP2 is stronger than that of TIRAP as a whole for PIP2 and that the short PI-binding motif (PBM) contributes to the affinity between PBD and PIP2. Four PIP2 molecules can be accommodated by distinct lysine-rich surfaces on the dimeric PBM. Along with the known PI-binding residues (K15, K16, K31, and K32), additional positively charged residues (K34, K35, and R36) showed strong affinity toward PIP2. Lysine-to-alanine mutations at the PI-binding residues abolished TIRAP’s affinity for PIP2; however, K34, K35, and R36 consistently interacted with PIP2 headgroups through hydrogen bond (H-bond) and electrostatic interactions. TIRAP exhibited a PIP2-analogous intermolecular contact and binding affinity toward PIP3, aided by an H-bond network involving K34, K35, and R36. The present study extends our understanding of TIRAP’s membrane association, which could be helpful in designing peptide decoys to block TLR2-, TLR4-, TLR7-, and TLR9-mediated autoimmune diseases.

## Introduction

The vertebrate innate immune system possesses germ-line encoded pattern recognition receptors (PRRs), which recognize conserved patterns of pathogenic components and initiate a protective response (1). Toll-like receptors (TLRs) are the most studied PRRs, which recognize exogenous pathogen-associated molecular patterns or endogenous damage-associated molecular patterns. Activation of TLRs results in homo- or heterodimerization-mediated recruitment of downstream adaptors to initiate complex cascades of signal transduction for the production of pro-inflammatory cytokines and interferons (2). The human genome encodes 10 functional TLRs, which are distributed on both the cellular (TLRs 1, 2, 4, 5, 6, and 10) and endosomal membranes (TLRs 3, 7, 8, and 9) (3). TLRs recognize a diverse variety of ligands, such as TLR1/2 recognizes triacyl lipopeptide (e.g., Pam3CSK4) (4); TLR2/6, diacyl lipopeptide (e.g., Pam2CSK4) (5); TLR3, viral double-stranded RNA (6); TLR4, lipopolysaccharide (LPS) (7); TLR5, bacterial flagellin (8); TLR7 and 8, viral single-stranded RNA (9, 10); and TLR9, bacterial single-stranded CpG DNA (11). The natural ligand for TLR10 has not yet been identified; however, some evidence suggests that it might recognize virus particles or TLR1/2 or TLR2/6 ligands (12–14).

Once activated, TLRs dimerize and recruit either myeloid differentiation primary response 88 (MyD88) protein or toll/interleukin 1 receptor (TIR) domain-containing adapter-inducing interferon β (TRIF) through their TIR domains. While MyD88 is the primary adaptor for most TLRs, only TLR3 and TLR4 recruit TRIF in a MyD88-independent manner. Thus, TLR4 can initiate signal transduction through both the MyD88 pathway and TRIF pathway, which occurs after internalization of cell surface-located TLR4 into the endosomal membrane (15). TLR2, TLR4, TLR7, and TLR9 essentially require TIR domain-containing adaptor protein (TIRAP), a bridging adaptor, which assists in recruiting MyD88 to the activated TLRs (16, 17). TIRAP exists as a physiological dimer while constitutively associated with phosphatidylinositol (PI) 4,5-bisphosphate (PIP2) on the cytoplasmic face of the cell membrane by means of a PI-binding motif (PBM; residues 15–35) present within the PI-binding domain (PBD; residues 1–40) (18, 19). In the endosomal membrane, PI (3,4,5) trisphosphate (PIP3) or possibly PI (3,5) P2 facilitates the anchoring function of TIRAP (16). The crystal structure of the TIRAP–TIR domain revealed that the N-terminal sequences harboring the PBD of both monomers face the same direction, supporting the membrane targeting mechanism of TIRAP (20, 21). Mutagenesis studies revealed that a stretch of basic residues, consisting mostly of lysine (K15, K16, K31, and K32), are crucial for anchoring TIRAP to PIP2-rich lipid rafts (18, 19). Chimeric TIRAP, with the PBD from phospholipase C δ1, retains its activity during LPS-mediated TLR4 signaling, while degradation of PIP2 with bacterial phosphatases deteriorates TIRAP’s membrane targeting activity (18). This indicates that the PBD plays a significant role in shaping the overall structure and function of TIRAP for effective signal transduction.

In recent years, TIRAP has been investigated intensively because of its importance in the signal transduction pathway of TLR4—the principal agent responsible for sepsis, an endotoxin-induced deadly autoimmune disease. Some evidence indicates that TIRAP could be an effective drug target to treat TLR2- and TLR4-mediated inflammatory diseases. In an interesting development, peptides derived from MyD88- or TLR-interacting surface patches of TIRAP blocked LPS-mediated signal transduction in mouse models of sepsis and rheumatoid arthritis (22). Furthermore, peptides derived from the TIR domain of TLR2 inhibited agonist-induced TLR2-, TLR4-, TLR7-, and TLR9-mediated aberrant autoimmune signaling by directly targeting TIRAP with a high affinity (23). Structural and biochemical investigations have provided several critical details of TIRAP’s physiological function as an upstream adaptor for MyD88. Although the crystal structure of the C-terminal TIR domain (residues 79–221) of TIRAP has been solved (20, 21), and an NMR structure of PBM (residues 15–35) has been recently reported (19), a complete view of full-length TIRAP (residues 1–221) along with its PI anchoring mechanism at the plasma/endosomal membrane remains elusive. A detailed atomic-level description of this mechanism is essential to understand the biophysicochemical nature of the different surfaces involved in MyD88, TLR, and PI interactions.

In the present study, we constructed a full-length molecular model of TIRAP using homology modeling, protein threading, and potential energy refinement approaches. The complete amino acid sequence of 221 residues was used in the model. Protein–protein docking was performed to predict an energetically favorable dimeric model mimicking the physiological organization of TIRAP. Molecular dynamics (MD) simulations over a pure and a PIP2-containing membrane bilayer were carried out to understand its role as a sorting adaptor for TLR2 and TLR4. A molecular mechanics/Poisson–Boltzmann surface area (MM/PBSA) method was performed to predict the binding affinity between different segments of TIRAP and PIP2. Furthermore, we studied the interaction of TIRAP with a PIP3-containing bilayer and compared that with the PIP2-containing bilayer. Altogether, our results provide a mechanistic insight into the structure and function of TIRAP, which could be utilized for designing specific peptides as decoys to inhibit aberrant TLR2-, TLR4-, TLR7-, and TLR9-mediated signaling.

## Materials and Methods

### Construction of the TIR Domain Dimer

The crystal structure of monomeric TIRAP–TIR (residues 79–221) was obtained from the Protein Data Bank (PDB) using the PDB ID: 3UB2 (20). However, the actual TIR domain spans residues 84–221 (UniProtKB accession number: P58753). The disordered AB loop was modeled in the SWISS-MODEL workspace (24) using 3UB2 as the template. The dimeric structure was obtained by performing protein–protein docking using the ZDOCK server (25). The residues reported to form the dimer interface in TIRAP–TIR (20, 21) were explicitly specified as binding residues during docking calculation. The remaining parameters were set to default. A total of 100 predicted complexes were generated, and the top ranked prediction was chosen based on the highest ZDOCK score (i.e., a statistical pair potential score of 1,564.68). Energy minimization was carried out using the GROMACS 5.1.4 simulation package (26) to relieve steric clashes between side chain and main chain atoms.

### Construction of the N-Terminal Domain (NTD) Dimer

The NTD (residues 1–78) was modeled using the threading-based I-TASSER modeling server (27). I-TASSER identifies the best templates by employing LOMETS threading programs based on the *Z*-score. The PDB IDs used for modeling were 5T7Q_A, 3J9A_A, 2MK0_A, 3ZIF_R, 2V6L_Z, 3J65_Q, and 2FFT_A. The letter after the underscore is the chain identifier. Among the top five models, the model having the highest *C*-score (i.e., −4.11) was selected for protein–protein docking. The dimeric state of the NTD was predicted using ZDOCK with default parameters and without specifying any binding or blocking residues. The top ranking docked conformation was optimized by energy minimization.

### Construction of the Full-Length TIRAP Dimer

The PBD dimer was oriented over the TIR dimer on a straight axis, such that the extreme N-terminal residue (S79) of the TIR domain and the extreme C-terminal residue (G78) of the NTD were within covalent bonding distance of each other. A peptide bond was then patched between those residues, followed by geometry optimization using Discovery Studio Visualizer 4.0 (DSV4.0; Dassault Systèmes, San Diego, CA, USA). Energy minimization was performed using GROMACS to optimize atomic conflicts. Loop modeling was performed for five residues upstream and downstream of the built peptide bond using MODLOOP server (28). The accuracy of the stereochemical parameters was checked after MD simulation as described in Section “Free Energy Landscape.”

### MD Simulations Using Pure Dipalmitoylphosphatidylcholine (DPPC) and DPPC–PI Membranes

Separate and concurrent MD simulations were performed for the dimeric TIRAP over a pure DPPC and a DPPC bilayer containing four PIP2/PIP3 molecules. For the TIRAP–DPPC system, the protein was oriented ~5 Å above N4 atoms of the upper leaflet of the bilayer. Similarly, the dimeric TIRAP was oriented over a PIP2-containing membrane, where the side chains of the PI-binding residues, K15, K16, K31, and K32 were at least 5, 10, or 15 Å away from the N4 atoms, producing three different simulation systems. Four phospholipids, aligned underneath the four lysine pairs [(K15–K16)_2_ and (K31–K32)_2_], were replaced by four PIP2s, followed by a round of energy minimization. The TIRAP–DPPC–PIP3 system was constructed by replacing PIP2 with PIP3. The PIP2 and PIP3 topologies were obtained from the automated topology builder server (29), which uses a quantum mechanics methodology to generate GROMACS-compatible topologies of novel molecules. A hybrid force field was constructed by combining Berger-lipid and GROMOS96-54A7 parameters for representing lipids and proteins, respectively (30). The simulation box was filled with simple point charge water and an appropriate amount of counter ions (either Na^+^ or Cl^−^). Energy minimization was performed using the steepest descent algorithm until a termination gradient of 1,000 kJ mol^−1^ nm^−1^ was reached. Temperature coupling (NVT) was performed using a V-rescale coupling scheme at a reference temperature of 300 K for 100 ps with positional restraints. Pressure equilibration (NPT) was carried out using a Parrinello–Rahman scheme at 1 bar for 1 ns with backbone restraints. For each system, production runs of 100 ns duration were performed without any backbone restraints using NPT ensemble. Short-range van der Waals and electrostatic interactions were calculated using a cutoff radius of 12 Å. Long-range electrostatics were handled using the particle mesh Ewald algorithm. Grid-based neighbor searching was performed using the Verlet scheme. All bonds were harmonically constrained using the LINCS method. Periodic boundary conditions were applied, and trajectories were saved every 2 ps. Data analysis was performed using XMgrace,^1^ VMD 1.9.2 (31), PyMOL 1.7 (Schrödinger, LLC, New York, NY, USA), DSV 4.0, and other analysis programs available in GROMACS. The area per lipid (APL) was calculated using GridMAT-MD (32).

### Binding Affinity Calculations

The binding free energies (BFE) between different segments of the TIRAP and PI molecules were calculated using the MM/PBSA method (33). In this approach, the free energy of binding between two species is estimated as follows:

(1) $$\begin{array}{l} \Delta G_{\text{bind}}=\langle G_{\text{complex}}\rangle -\langle G_{\text{protein}}\rangle -\langle G_{\text{ligand}}\rangle , \end{array}$$

where Δ*G*_bind_ is the total BFE and remaining components are the free energy of the complex, the protein, and the ligand. The free energy of each component is computed as follows:

(2) $$\text{G}=G_{\text{bond}}+G_{\text{ele}}+G_{\text{vdW}}+G_{\text{pol}}+G_{\text{npol}}-\text{TS},$$

where *G*_bond_ (bonded, angle, and dihedral), *G*_ele_, and *G*_vdW_ are the standard molecular mechanics energy terms derived from bonded, electrostatic, and van der Waals interactions, respectively. *G*_pol_ and *G*_npol_ are the polar and non-polar contribution to the solvation free energies. The polar contribution is obtained by the Poisson–Boltzmann equation and the non-polar contribution is calculated using a linear relation to the solvent accessible surface area method. The final term, TS, is the entropic contribution (absolute temperature *T* multiplied by entropy *S*), which is estimated by a normal mode analysis. We used the “g_mmpbsa” tool (34) to calculate the BFE, considering 1,000 structural frames between 80 and 100 ns of the MD trajectory.

### Free Energy Landscape (FEL)

The FEL was computed to visualize different free energy states attained by TIRAP during the course of membrane association. The radius of gyration (Rg) and root mean square deviation (RMSD) were variables used for calculating FEL with the “sham” tool in GROMACS. The landscape was visualized using a demo version of Mathematica 11.2 software (Wolfram Research, Inc., Champaign, IL, USA, 2017). Several structures were extracted from the low-energy region of the plot using “get_timestamp.py” script,^2^ and one representative structure from the equilibrated portion of dynamics trajectory was selected to evaluate the stereochemical accuracy using the Rampage (35) and ProSA-Web servers (36).

### Essential Dynamics

Principal component analysis, or essential dynamics, was performed to extract structural coordinates associated with the principal component of each simulation system. Such coordinates give a general representation of the global low-frequency molecular movement during the MD simulation. A covariance matrix was constructed containing the main chain atoms of TIRAP. Diagonalization of the matrix resulted in a set of eigenvectors and their corresponding eigenvalues. The first eigenvector, also called the principal component, usually contains the most dominant motion in the trajectory. Porcupine plots were constructed using the “modevectors.py”^3^ script to display the differential displacements of TIRAP on pure DPPC and DPPC–PI membranes.

### Determination of the Electrostatic Potential Surface

The electrostatic potential surface around TIRAP was calculated using the PyMOL-based “apbsplugin.py” tool.^4^ The linearized Poisson–Boltzmann equation was used with a solvent radius of 1.4 Å. The positive and negative isosurfaces were visualized with a contour (kT/e) value of 1.

## Results

### The Overall Structure of TIRAP Reveals Key Features for Membrane Association

**Figure 1:**
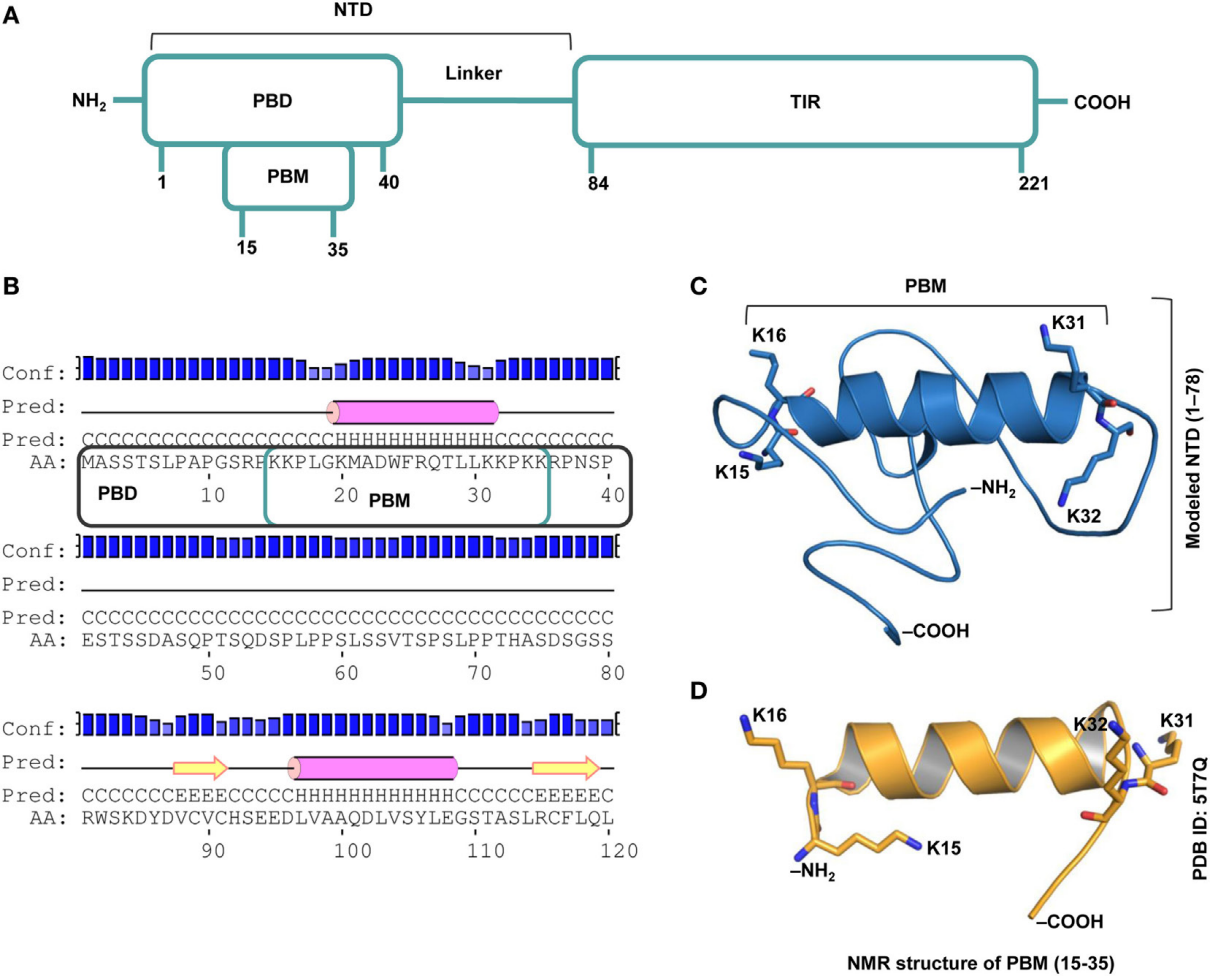
Overall topology of the toll/interleukin 1 receptor (TIR) domain-containing adaptor protein (TIRAP). **(A)** The overall domain organization of TIRAP. Amino acid positions are indicated at the bottom of each segment. **(B)** The predicted secondary structure of residues 1−120 showing the random coiled nature of the N-terminal domain (NTD; residues 1−83). The phosphatidylinositol (PI)-binding motif (PBM; residues 15−35) and the PI-binding domain (PBD; residues 1−40) are enclosed in teal and black boxes, respectively. **(C)** A three-dimensional model of the NTD (residues 1−78) showing the PI-binding residues: K15 and K16 at one end and K31 and K32 at the other end of the α helix (residues 17−31). Although NTD spans residues 1–83, we modeled only residues 1–78, as residues 79–221 are present in the crystal structure of the TIR domain (PDB ID: 3UB2). **(D)** The NMR-derived structure of PBM (PDB ID: 5T7Q; residues 15−35).

TIRAP consists of a C-terminal TIR domain that bridges the interaction between MyD88 and TLR and an NTD that contains the PBD and the PBM, which are essential for PI binding on the cytoplasmic side of the plasma/endosomal membranes (Figure 1A). While the PBD is attributed to membrane association and localization, the PBM is required for membrane targeting and direct binding to PI in lipid rafts (18). Recently, using an NMR structure of the PBM and site-directed mutagenesis, it was confirmed that four positively charged residues in each monomer (K15, K16, K31, and K32) are crucial for PIP2 binding and membrane localization (19). Our prediction of the secondary and tertiary structures of NTD (Figures 1B,C) clearly supports the helical nature of the PBM structure derived by NMR (Figure 1D). The modeled NTD (residues 1−78) is mostly a random coil with a single α helical segment between residues 17 and 31. Since the modeling server utilized the NMR structure of PBM (PDB ID: 5T7Q) as one of the templates [see Construction of the N-Terminal Domain (NTD) Dimer], we obtained an exact structure for residues 15–35. The flexible regions at both ends of the helix recognize PI molecules. All basic residues of the PBM face the same direction, which is a property required for electrostatic binding to acidic PI headgroups.

**Figure 2:**
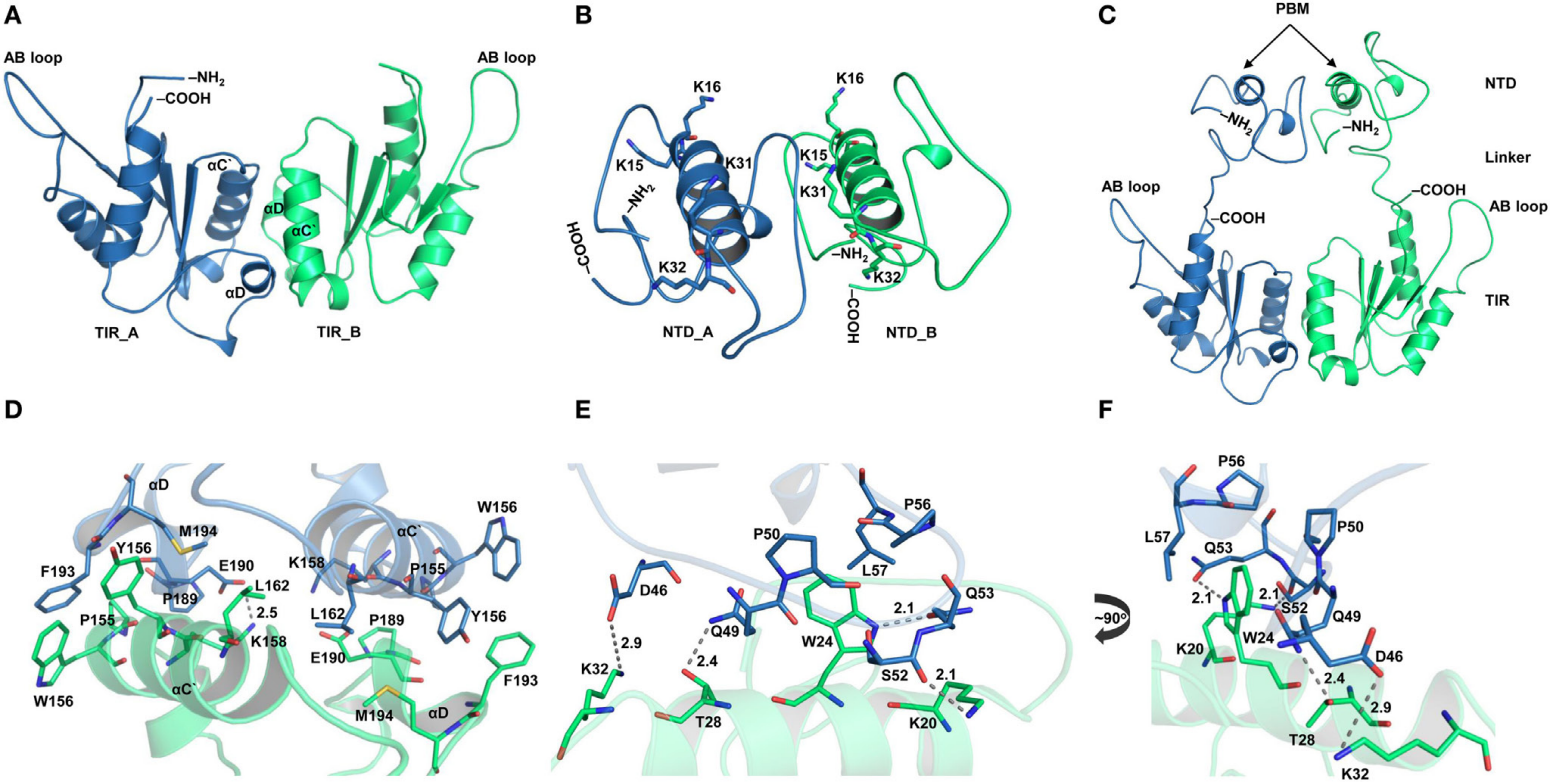
Structural organization of individual domains of toll/interleukin 1 receptor (TIR) domain-containing adaptor protein (TIRAP). **(A)** The overall structure of a modeled TIR domain dimer. **(B)** A predicted model of the N-terminal domain (NTD). **(C)** Initial model (energy minimized) of a full-length TIRAP with the NTD and TIR domains arranged in a physiological dimeric condition. **(D)** A closer view of the dimer interface between TIR monomers involving residues from αC′ and αD helices. **(E)** A detailed view of the NTD dimerization, including residues from both phosphatidylinositol (PI)-binding motif (PBM) and PI-binding domain (PBD). **(F)** A 90° rotated view of panel **(E)** for clarity. Dashed lines represent hydrogen bonds, and digits indicate distances in angstrom units.

TIRAP has been reported to exist as a physiological dimer constitutively associated with the plasma/endosomal membranes. We constructed a dimeric model of TIRAP to better understand its structure and function. We performed TIR domain dimerization using protein–protein docking based on the dimer packing information available from the X-ray crystallographic structure (PDB ID: 3UB2). The dimer packing is mainly governed by amino acids belonging to the αC′ and αD helices of both monomers (Figures 2A,D). However, due to the lack of relevant information, the dimeric NTD was obtained *via* an automated protein–protein docking approach without specifying any potential binding or blocking residues (Figure 2B). PBMs of both monomers pack against each other through numerous intermolecular hydrogen bonds (H-bonds) and hydrophobic interactions (Figures 2E,F). Specifically, W24 stacks itself inside a hydrophobic pocket created by residues P50, P56, and L57. The side-chain amino groups of K20 and K32 form H-bonds with the backbone carbonyl group of Q53 and the side-chain carboxyl group of D46, respectively. T28, which is crucial for TIRAP phosphorylation by interleukin-1 receptor-associated kinase (IRAK) 1 and IRAK4 (37), forms an H-bond with the side-chain amino group of Q49. Notably, both PBMs in the dimeric NTD show a parallel orientation separated by a protruding loop, and the four PI-binding residues are exposed in the same direction. This arrangement provides TIRAP with the necessary surface area for anchoring to discrete PI molecules on the plasma membrane. Thus, the NTD dimer supports the previously assumed physiological orientation of TIRAP, based on the analysis of isolated PBM or TIR structures (16, 18–21). The full-length model of TIRAP contains a long and flexible linker (residues 41−83) that spans between the PBD and the TIR domains (Figure 2C).

### Full-Length TIRAP Dimer Shows Differential Dynamics over the DPPC Bilayer

**Figure 3:**
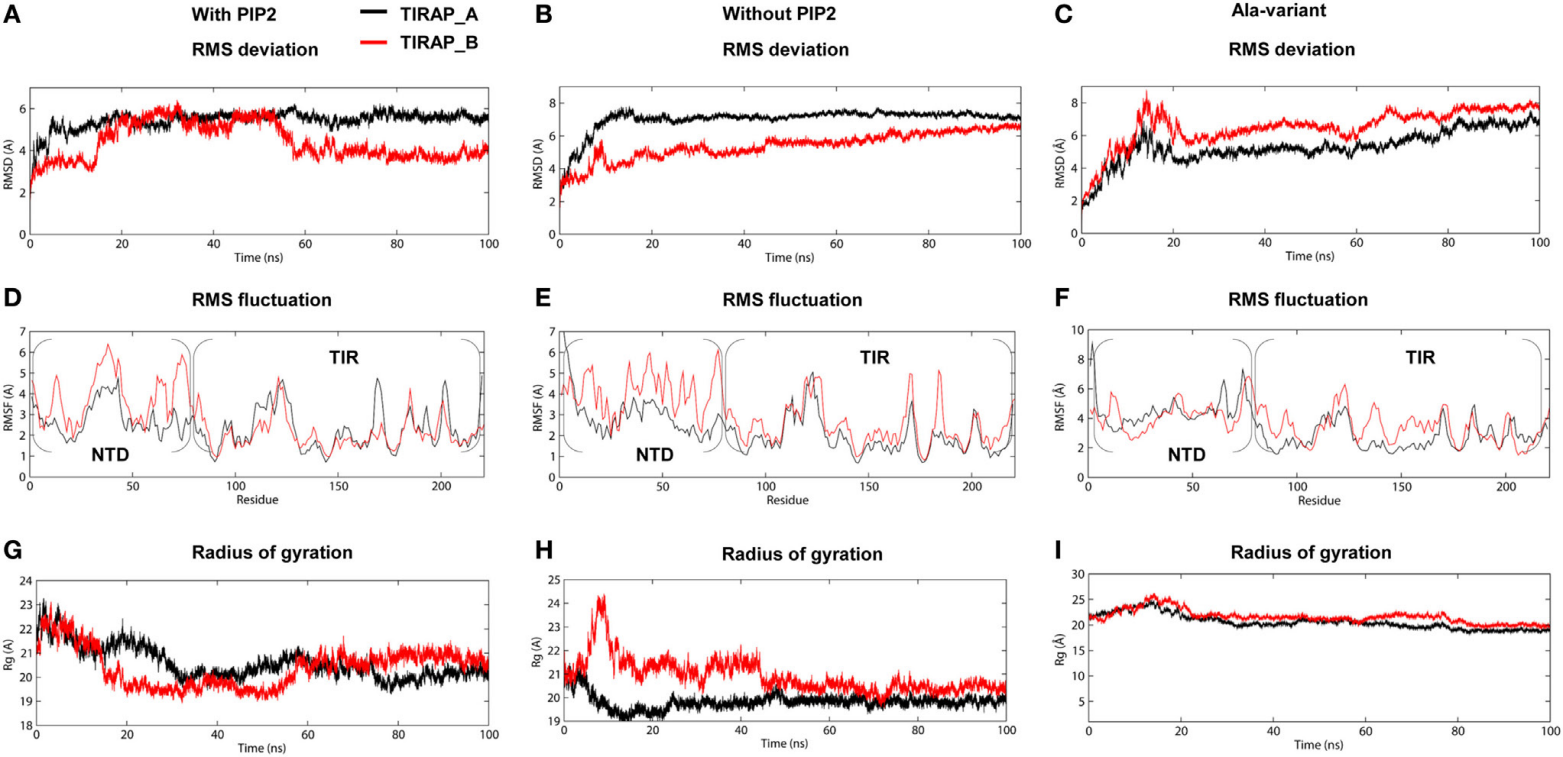
Stability parameters of toll/interleukin 1 receptor domain-containing adaptor protein (TIRAP) as a function of simulation time. **(A–C)** Root mean square deviation; **(D–F)** root mean square fluctuation; and **(G–I)** radius of gyration. The left and center columns contain stability parameters for phosphatidylinositol 4,5-bisphosphate (PIP2)-bound and unbound TIRAPs, respectively, while the right column contains stability parameters for a mutant TIRAP (K15 → A, K16 → A, K31 → A, and K32 → A). Also, note that each plot shows parameters for chain **(A)** (black) and chain **(B)** (red).

The TIRAP–PBM was found to be intrinsically disordered in solution, but gained α helicity in the presence of micellar phospholipids (19). This indicates that membrane phospholipids are essential for the physiological behavior of PI-binding residues and PBM as a whole. We carried out extensive MD simulations with TIRAP by placing it over a pure and a PIP2-containing DPPC bilayer to differentiate its folding behavior in the presence and absence of PIP2. Both of these simulations were performed multiple times with increasing distance between TIRAP and DPPC (Table 1). The system in which TIRAP was ~5 Å above the PIP2-containing membrane had the most stable dynamics during the MD simulation (Figure S1 in Supplementary Material). In the presence of PIP2, the protein showed a consistent RMSD of backbone atoms, achieving stability soon after 60 ns in the MD simulation (Figure 3A). On the other hand, TIRAP exhibited continuous structural evolution over the pure DPPC without reaching equilibration during 100 ns (Figure 3B). This indicates that PIP2 not only provides a platform for TIRAP’s membrane localization but also assists in its structural stability and physiological folding behavior. Comparison of local fluctuations at the residue level revealed that the TIR domains of both PIP2-bound and unbound protein display a similar trend (Figures 3D,E); however, the NTD’s fluctuation was different. The conformational flexibility of the NTD is reasonable, as it contains mainly random coiled segments (Figures 1B and 2B). An Rg plot indicated that PIP2-bound and only lipid-bound TIRAPs converged to a common point at the end of 100 ns of MD simulation, suggesting that the protein gained a compact three-dimensional structure irrespective of the solvent environment (Figures 3G,H).

**Table 1:** The molecular composition of different MD simulations performed in our study.

Bilayer	No. of lipids	No. of waters	No. of PIs	No. of counter ions	Distance of TIRAP from the membrane surface (Å)	Total no. of molecules (excluding protein)
DPPC	284	18,825	4 (PIP2)	18 Na^+^	5	19,131
DPPC	284	24,537	4 (PIP2)	18 Na^+^	10	24,843
DPPC	284	32,537	4 (PIP2)	18 Na^+^	15	32,843
DPPC	288	25,592	–	2 Cl^−^	5	25,882
DPPC	288	25,515	–	2 Cl^−^	5	25,805
DPPC	284	22,834	4 (PIP3)	26 Na^+^	5	23,148
DPPC^a^	284	22,854	4 (PIP2)	26 Na^+^	5	23,168

*All simulations were performed for a duration of 100 ns*.

*^a^MD simulation system containing the lysine-to-alanine mutant of TIRAP*.

*DPCC, dipalmitoylphosphatidylcholine; MD, molecular dynamics; PI, phosphatidylinositol; PIP2, PI 4,5-bisphosphate; PIP3, PI (3,4,5)-trisphosphate; TIRAP, toll/interleukin 1 receptor domain-containing adaptor protein*.

**Figure 4:**
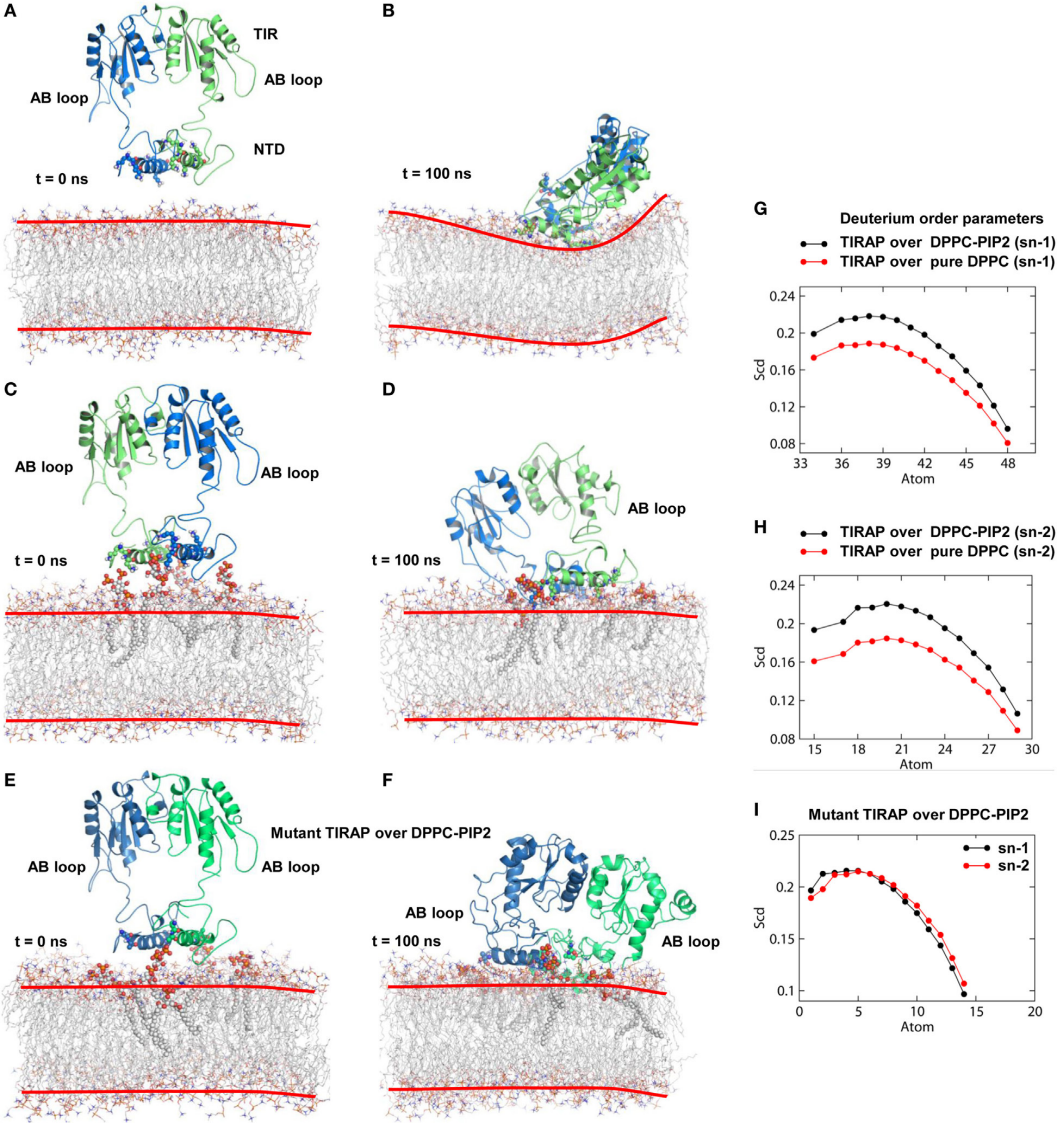
Molecular dynamics (MD) simulation of toll/interleukin 1 receptor domain-containing adaptor protein (TIRAP) over different dipalmitoylphosphatidylcholine (DPPC) bilayers. **(A)** Starting model of TIRAP over a pure DPPC bilayer. **(B)** The last snapshot of TIRAP (*t* = 100 ns) over the DPPC-only bilayer. The curvature of the membrane surface is marked with red lines for clarity. **(C)** TIRAP model over a DPPC–phosphatidylinositol (PI) 4,5-bisphosphate (PIP2) bilayer. The PIP2 and PI-binding residues are represented as solid spheres. **(D)** TIRAP model after 100 ns of MD simulation over a DPPC–PIP2 bilayer. **(E)** Initial model of the mutant TIRAP (K15 → A, K16 → A, K31 → A, and K32 → A) over the DPPC–PIP2 bilayer. **(F)** 100 ns snapshot of the mutant TIRAP. In each panel, chain A is colored blue and chain B is green. **(G)** Deuterium order parameter of sn-1 chains of DPPC lipids. **(H)** Deuterium order parameter for sn-2 chains. In both G and H, black lines represent order parameters of PIP2-bound DPPC, while red lines represent order parameters of pure DPPC lipids. **(I)** Order parameters of DPPC–PIP2 bilayer under the mutant TIRAP. Black and red lines represent sn-1 (carbons 34–50) and sn-2 (carbons 15–31) chains, respectively.

Physiologically, TIRAP is explicitly found at the leading edge of the membrane (in fibroblasts) or at membrane ruffles (in macrophages) (38), which are rich in PIP2 and actin filaments. In addition to PIP2 molecules, TIRAP closely associated with the actin filaments of the membrane (18). These regions of the membrane usually provide anchoring support to proteins from various cellular pathways. We found that TIRAP induces profound curvature on a pure DPPC bilayer (Figures 4A,B), allowing large rotational and translational movements of acyl chains with respect to the bilayer normal (Figures 4G,H). On the other hand, the PIP2-containing bilayer maintained membrane integrity, with less curvature (Figures 4C,D) and a physiologically relevant lipid order parameter (39), i.e., −*S*_CD_ = ~0.20 at carbon atoms 15–21 of sn-2 and 34–42 of sn-1 chains of the bilayer (Figures 4G,H). The average APL in the pure DPPC membrane (61.32 Å^2^) was greater than that in the DPPC–PIP2 bilayer (60.26 Å^2^). Although both of these values are consistent with previous NMR experiments (with the APL varying from 56 to 72 Å^2^) (40–42), the 1.06 Å^2^ shrinkage in APL indicates a PIP2-induced effect. Moreover, the APL of the upper leaflet of DPPC–PIP2 (59.41 Å^2^) was 1.91 Å^2^ less than that of pure DPPC (61.32 Å^2^), while the lower leaflets of both systems each had an APL = ~61.20 ± 0.5 Å^2^. TIRAP also had different dynamics in two different solvent environments. PIP2-anchored TIRAP displayed an ordered smooth movement, as shown by the direction and length of spikes in the porcupine plot (Figures S2A,B in Supplementary Material). The TIR domain gradually moved toward the PBD, while the PBD moved *vice versa*. Remarkably, the opposite movements of both domains conserved all necessary features of dimerization, the orientation of the PI-binding residues, and the overall topology of the PBD. In the final MD snapshot, the PBD and TIR domains gained a well-organized, compact molecular architecture. However, TIRAP’s movement in pure DPPC was random, in that the TIR and PBD moved in all directions. Moreover, although TIRAP achieved a compact molecular structure at the end of MD simulation, the PI-interacting residues in the PBM were poorly organized with respect to the membrane.

To observe the effect of mutation of the PI-binding residues on the structure of TIRAP, we carried out a 100 ns MD simulation of a TIRAP mutant (K15 → A, K16 → A, K31 → A, and K32 → A) over the DPPC–PIP2 bilayer. We found that the mutant failed to reach an equilibrium plateau during the simulation (Figure 3C). The root mean square fluctuation plot showed an asymmetrical fluctuation of NTD residues compared to the wild-type protein on both DPPC and DPPC–PIP2 membranes (Figure 3F). Although the Rg values remained consistent (Figure 3I), the overall tertiary fold was relatively less compact than that of the wild-type. This was also revealed by the porcupine plot where the mutant TIRAP had a high-amplitude movement in all directions (Figure S2C in Supplementary Material), which correlates with the elevated RMSD curves of both subunits. However, the membrane maintained its integrity by curving less (Figures 4E,F), with an −*S*_CD_ value of ~0.20 at carbon atoms 15–21 of sn-2 and 34–42 of sn-1 chains (Figure 4I), and an average APL of 60.22 and 61.95 Å^2^ in the top and bottom leaflets of the bilayer, respectively.

### The Full-Length TIRAP Shows Considerable Stereochemical Accuracy and Dimer Packing Interactions

Several low energy structures were extracted from the Gibbs FEL to evaluate the stereochemical accuracy of PIP2-bound TIRAP (Figure S3A in Supplementary Material). The Ramachandran plot of one representative frame (*t* = 75.312 ns) indicated that 97.7 and 97.3% of the residues from chains A and B, respectively, fall under the favored and allowed regions of the plot (Table S1 and Figures S4A–D in Supplementary Material). ProSA *Z*-scores of −5.8 and −5.79 for chains A and B, respectively, indicated that the modeled TIRAP has the structural quality of an X-ray crystal structure (Table S1 and Figures S4E–H in Supplementary Material). The model was further validated by observing the interaction between residues that form a dimer interface in the crystal structure of the isolated TIR domain. In particular, K158 of one monomer forms an H-bond with E190 of the other. Y159 of one monomer is situated close to M194 of the other due to hydrophobic attraction. L162 of both monomers face toward each other, forming hydrophobic interactions. P189 and F193 of one monomer pack against P155 and W156 of the other (20). These interactions were completely conserved in our full-length TIRAP dimer, with the exception of a salt bridge instead of an H-bond between K158 and E190 (Figures S3C–F in Supplementary Material). Thus, our lowest energy structure of TIRAP not only possesses considerable stereochemical accuracy but also displays the physiological orientation of key interfacial residues.

### PIP2 Has Greater Affinity for PBD than for the PBM and Whole TIRAP

Computational BFE provides a general estimate of binding affinity between two given molecules. We calculated the BFE between PIP2 and different segments of TIRAP using 1,000 frames extracted from the last 20 ns of the MD trajectory (Table 2). The total BFE of the TIRAP–PIP2 complex was −3,991.15 kJ mol^−1^, whereas those of PBD–PIP2 and PBM–PIP2 were −11,403.95 and −8,263.84 kJ mol^−1^, respectively. This indicates PIP2 has a stronger affinity for PBD than for PBM and TIRAP as a whole. The increased binding strength of the PBD–PIP2 complex could be due to the presence of additional basic amino acids residing outside of the PBM (e.g., R36). Decomposition of the BFE into individual energy terms revealed that electrostatic energy is the dominant contributor to the affinity between PIP2 and all individual segments analyzed. Further comparison of BFE between PIP2 and monomeric PBD or PBM (PBD_A-PIP2, PBD_B-PIP2, PBM_A-PIP2, and PBM_B-PIP2) showed a similar trend to that observed for the dimeric segments. The monomeric PBDs bind to PIP2 stronger than the monomeric PBMs. We concluded that PBD plays a greater role in PIP2 recognition and binding than the PBM for TIRAP’s membrane association. However, previous studies have shown that isolated PBM alone is sufficient to recognize and bind to PIP2 (18, 19). Interestingly, TIRAP has a greater affinity for PIP2 than for DPPC–PIP2 together. This could be explained by the fact that a large portion of TIRAP and DPPC do not participate in the interfacial interaction and, thus, their energetic contribution is null during the BFE calculation.

**Table 2:** Binding free energy (kJ mol^−1^) between PIP2 and important segments of TIRAP.

Complex groups	Δ_vdW_^a^	Δ_elec_^b^	Δ_ps_^c^	Δ_SASA_^d^	Δ*G*_Total_^e^
TIRAP–PIP2	−106.55 ± 23.89	−5,751.17 ± 199.80	1,891.38 ± 152.91	−24.81 ± 4.93	−3,991.15 ± 144.81
TIRAP–DPPC–PIP2	−1,079.77 ± 68.97	−10,873.77 ± 417.45	12,598.60 ± 720.47	−818.40 ± 15.78	−173.35 ± 544.98
PBD–PIP2	−97.04 ± 23.58	−13,184.38 ± 200.68	1,901.96 ± 152.70	−24.48 ± 2.99	−11,403.95 ± 112.04
PBD–DPPC–PIP2	−638.74 ± 59.82	−17,010.45 ± 473.75	11,483.39 ± 964.11	−783.69 ± 15.49	−6,949.49 ± 892.27
PBM–PIP2	−38.35 ± 15.11	−9,535.25 ± 147.37	1,323.40 ± 107.40	−13.64 ± 2.08	−8,263.84 ± 99.65
PBM–DPPC–PIP2	−269.17 ± 49.67	−12,876.59 ± 329.12	6,142.39 ± 933.46	−749.13 ± 14.85	−7,752.50 ± 952.17
PBD_A–PIP2	−35.89 ± 14.05	−4,067.33 ± 70.63	940.48 ± 71.87	−10.51 ± 1.80	−3,173.26 ± 36.97
PBD_B–PIP2	−54.62 ± 17.99	−4,017.56 ± 155.16	884.35 ± 131.24	−12.00 ± 2.40	−3,199.84 ± 61.24
PBM_A–PIP2	−11.72 ± 7.53	−3,048.29 ± 58.05	617.80 ± 63.31	−5.28 ± 1.49	−2,447.49 ± 35.62
PBM_B–PIP2	−25.05 ± 12.80	−2,932.35 ± 102.40	715.32 ± 80.59	−8.53 ± 1.62	−2,250.61 ± 50.85

*^a^Van der Waals energy*.

*^b^Electrostatic energy*.

*^c^Polar solvation energy*.

*^d^Solvent accessible surface area energy*.

*^e^Total binding free energy*.

*DPCC, dipalmitoylphosphatidylcholine; PBD, phosphatidylinositol (PI)-binding domain; PBM, PI-binding motif; PIP2, PI 4,5-bisphosphate; TIRAP, toll/interleukin 1 receptor domain-containing adaptor protein*.

To observe the mutational effect of PI-binding residues on the PIP2 binding affinity of TIRAP, we calculated the BFE between the Ala-variant of TIRAP (K15 → A, K16 → A, K31 → A, and K32 → A) and the DPPC–PIP2 bilayer (Table 3). Mutations of PI-binding residues completely abolished the affinity of TIRAP for PIP2 (Δ*G* = 889.99 kJ mol^−1^) and DPPC–PIP2 complex (Δ*G* = 4,486.48 kJ mol^−1^). Although the dimeric/monomeric PBD/PBM had milder binding affinities for PIP2 or DPPC–PIP2, these affinities were at least twofold less than those of the wild-type variant. This suggests that the affinity of PBD/PBM for PIP2 could be a result of the interaction between the additional positively charged residues K34, K35, and R36 and PIP2. We found that, even after mutation at PI-binding residues, PIP2 had a greater affinity for PBD than for PBM. This highlights the importance of K34, K35, and R36, which are present at the C-terminal end of PBM.

**Table 3:** Binding free energy (kJ mol^−1^) between PIP2 and important segments of TIRAP alanine variants (K15 → A, K16 → A, K31 → A, and K32 → A).

Complex groups	Δ_vdW_^a^	Δ_elec_^b^	Δ_ps_^c^	Δ_SASA_^d^	Δ*G*_Total_^e^
TIRAP–PIP2	−127.32 ± 22.40	−184.07 ± 18.34	1,226.61 ± 134.61	−25.22 ± 5.69	889.99 ± 15.44
TIRAP–DPPC–PIP2	−1,110.32 ± 59.62	−3,308.31 ± 151.04	9,713.91 ± 106.90	−808.77 ± 19.06	4,486.48 ± 108.31
PBD–PIP2	−102.86 ± 18.45	−7,816.85 ± 130.61	1,154.38 ± 107.25	−20.81 ± 2.71	−6,786.14 ± 82.80
PBD–DPPC–PIP2	−597.99 ± 40.01	−9,474.26 ± 204.82	7,611.79 ± 837.17	−765.72 ± 16.45	−3,226.19 ± 823.93
PBM–PIP2	−62.51 ± 14.44	−4,394.85 ± 51.91	863.41 ± 47.51	−14.43 ± 1.75	−3,608.39 ± 45.44
PBM–DPPC–PIP2	−402.09 ± 39.72	−6,264.13 ± 129.56	3,972.11 ± 705.21	−752.17 ± 16.73	−3,446.28 ± 693.40
PBD_A–PIP2	−39.89 ± 13.81	−2,494.39 ± 42.45	635.30 ± 59.81	−10.80 ± 1.84	−1,909.78 ± 33.89
PBD_B–PIP2	−61.23 ± 11.88	−2,399.45 ± 54.60	523.20 ± 63.37	−9.97 ± 1.92	−1,947.45 ± 31.97
PBM_A–PIP2	−17.20 ± 10.31	−1,457.93 ± 26.16	515.62 ± 27.89	−7.15 ± 1.36	−966.66 ± 32.58
PBM_B–PIP2	−44.07 ± 10.79	−1,392.06 ± 33.92	397.65 ± 25.89	−7.71 ± 1.29	−1,046.20 ± 24.06

*^a^Van der Waals energy*.

*^b^Electrostatic energy*.

*^c^Polar solvation energy*.

*^d^Solvent accessible surface area energy*.

*^e^Total binding free energy*.

*DPCC, dipalmitoylphosphatidylcholine; PBD, phosphatidylinositol (PI)-binding domain; PBM, PI-binding motif; PIP2, PI 4,5-bisphosphate; TIRAP, toll/interleukin 1 receptor domain-containing adaptor protein*.

### PIP2 Molecules Interact with Distinct Basic Patches on the PBM

**Figure 5:**
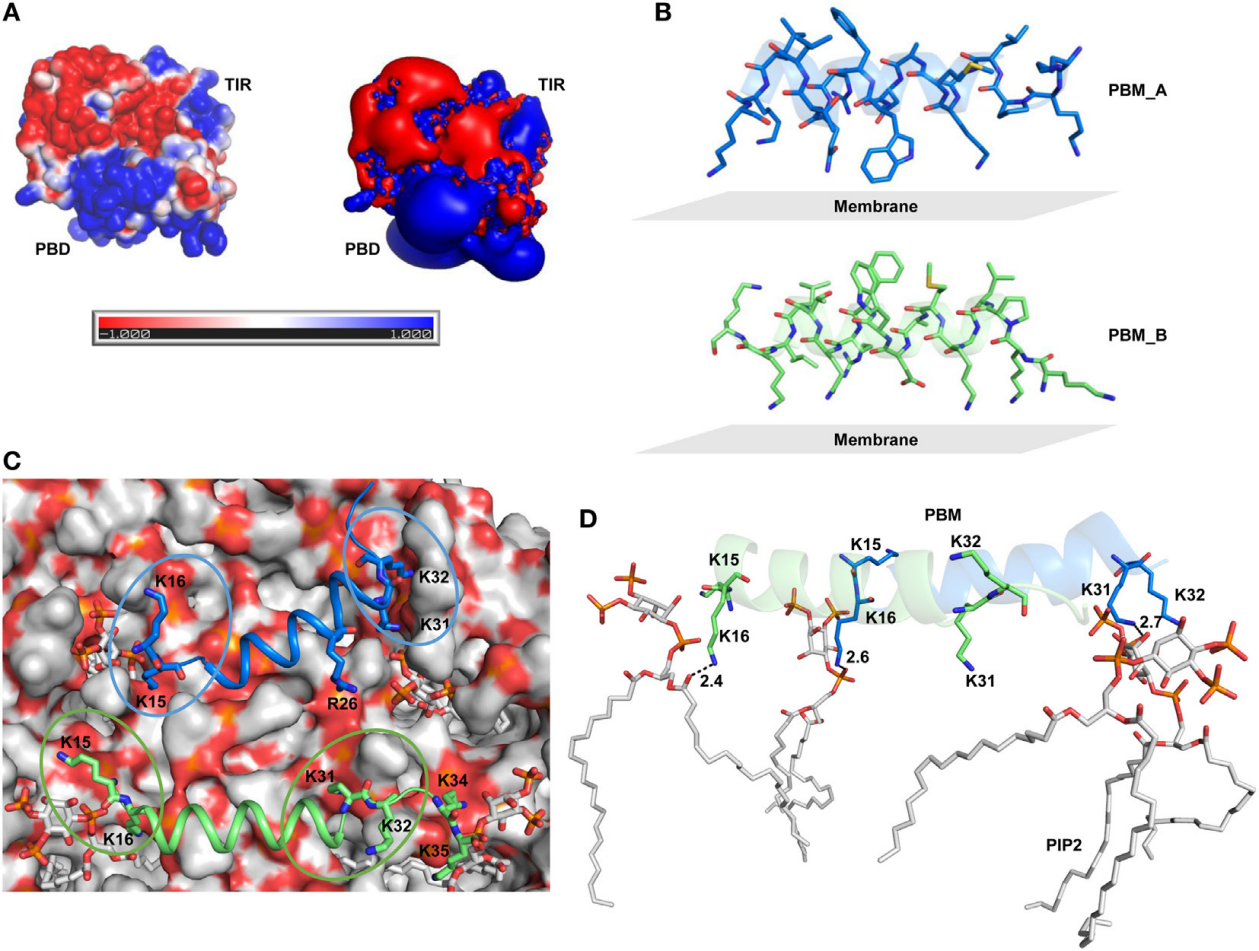
Surface electrostatics of toll/interleukin 1 receptor domain-containing adaptor protein (TIRAP) and the location of phosphatidylinositol (PI) 4,5-bisphosphate (PIP2) on the membrane surface. **(A)** Two different representations of electrostatic potential over TIRAP. The left image shows the positive (blue), negative (red), and neutral (white) surfaces of TIRAP, while the right image represents an electrostatic isosurface, showing only the surface distribution of the most positively and negatively charged residues on TIRAP’s surface. **(B)** Orientation of the PI-binding motif (PBM) side chains with respect to the membrane surface in the final snapshot after 100 ns of molecular dynamics (MD) simulation. **(C)** Top view of the membrane surface showing the position of PIP2 and PI-binding residues of TIRAP. **(D)** Intermolecular interaction between dimeric PBM and four PIP2 molecules at the end of 100 ns of MD simulation. Dashed lines represent hydrogen bonds, and digits indicate distances in angstrom units.

**Figure 6:**
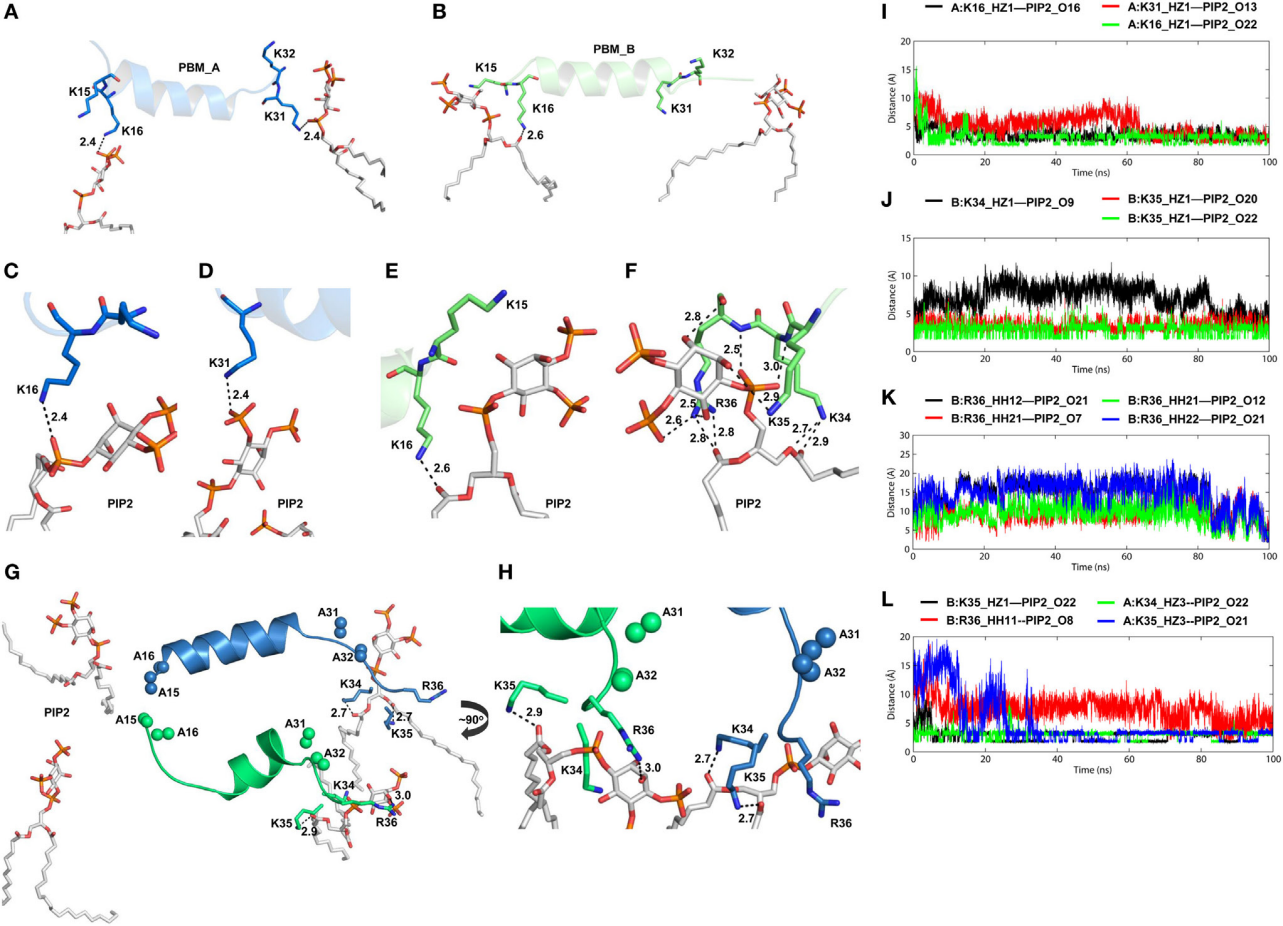
Intermolecular interactions between phosphatidylinositol (PI) 4,5-bisphosphate (PIP2) and positively charged residues of toll/interleukin 1 receptor domain-containing adaptor protein (TIRAP). Interaction of PIP2 with the PI-binding residues from **(A)** chain A and **(B)** chain B. **(C–E)** Close-up views of hydrogen bonds (H-bonds) formed between PIP2 and PI-binding residues. **(F)** One PIP2 trapped within a strong H-bond network formed by closely spaced K34, K35, and R36. **(G)** Interaction of PIP2 with TIRAP-PI-binding motif (PBM) with mutations K15 → A, K16 → A, K31 → A, K32 → A. Alanine residues are shown as sphere models, with blue for chain A and green for chain B. **(H)** A ~90° rotated view of panel **(G)** with close-up for clarity. Dashed lines represent H-bonds, and digits indicate distances in angstrom units. **(I)** H-bond distances between three PIP2-binding residues and PIP2. **(J)** Distance of H-bonds formed between K34 and K35 and PIP2. **(K)** H-Bond distances between atoms of R36 and the PIP2 headgroups. **(L)** Distance between interacting atoms of K34, K35, R36, and PIP2 in a mutant TIRAP as a function of time.

**Figure 7:**
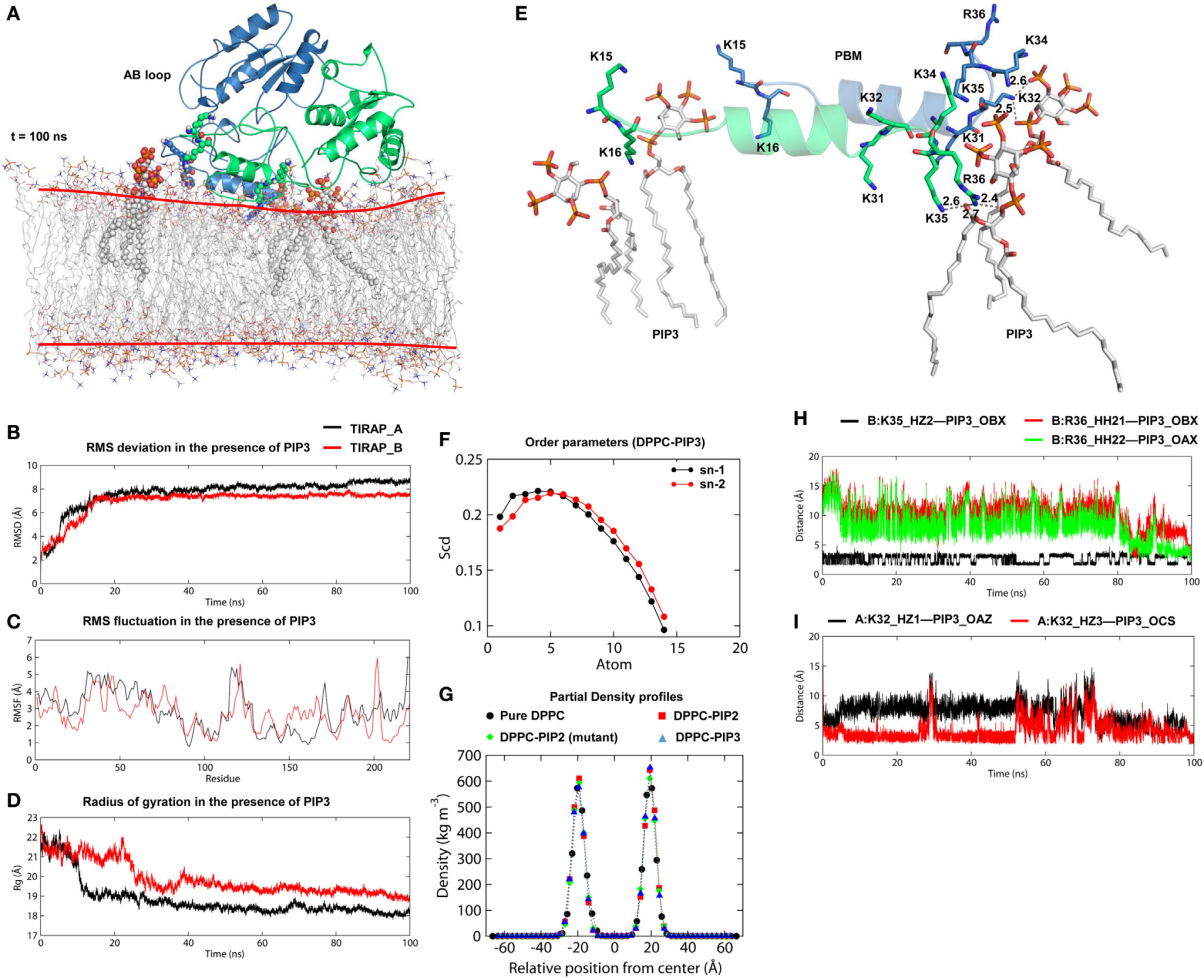
Stability and interaction properties of toll/interleukin 1 receptor domain-containing adaptor protein (TIRAP) and a dipalmitoylphosphatidylcholine (DPPC)-phosphatidylinositol (PI) (3,4,5)-trisphosphate (PIP3) bilayer. **(A)** Overall structure of TIRAP over a DPPC–PIP3 bilayer. TIRAP is colored blue for chain A and green for chain B. The red lines on the membrane indicate the approximate positions of DPPC headgroups. PIP3 and PI-binding residues are shown as spheres. **(B)** Root mean square deviation; **(C)** root mean square fluctuation; and **(D)** radius of gyration (Rg). The black and red curves represent chains A and B, respectively. **(E)** Interaction of PIP3 with TIRAP–PBM. Dashed lines represent hydrogen bonds, and digits indicate distances in angstrom units. **(F)** Order parameters for sn-1 and sn-2 chains of DPPC lipids in the presence of PIP3. **(G)** Headgroup density profiles of four different DPPC membranes: black: pure DPPC, red: DPPC–PI 4,5-bisphosphate (PIP2), green: DPPC–PIP2 (under mutant TIRAP), and blue: DPPC–PIP3. **(H,I)** Intermolecular distances between H-bond-forming atoms of K32, K35, R36, and PIP3 as a function of time.

TIRAP lacks a definite transmembrane segment for membrane attachment. Instead, it solely depends on its PBM and positively charged residues to associate with the cell membrane. As in other PIP2-binding proteins, TIRAP’s PBD contains an extensive basic surface consisting of lysine and arginine (Figure 5A). The strong positive charge and long flexible side chains of these residues face the negatively charged membrane phospholipids (Figure 5B). This electrostatic complementarity allows for an energetically favorable membrane association with TIRAP, until its phosphorylation-mediated ubiquitination and degradation. Previous reports indicate that one monomer of TIRAP carries two distinct PI-binding sites on the flexible regions of the N-terminal PBM. Thus, dimeric TIRAP should contain four binding sites, where the sites of opposite monomers are more closely spaced than those of the same monomer (Figure 5C). We performed MD simulations by placing PI-binding residues 5 Å apart from PIP2 molecules over a DPPC-PIP2 bilayer. Three H-bonds were observed between the oxygen atoms of PIP2 headgroups and the amino groups of K16 of both PBMs and K31 of PBM_A (Figure 5D), while K15 of both PBMs and K32 of PBM_A are spaced ~5 Å from PIP2 headgroups, indicating an electrostatic interaction. In our simulation, one PIP2 molecule completely lost contact with K31 and K32, but was entangled in a stronger H-bond network with K34, K35, and R36 (Figures 6A–F). This indicates that the neighboring positively charged residues could also play important roles for PIP2-dependent membrane anchoring of TIRAP. The porcupine plot suggested that the displacement of K31 and K32 away from a single PIP2 molecule could be due to the greater movement of chain B (green monomer) observed during the MD simulation (Figure S3B in Supplementary Material). Calculation of H-bond distances as a function of time revealed that the PI-binding residues interacted consistently with PIP2 throughout the 100 ns MD simulation (Figure 7G). A stronger interaction network was observed between PIP2 and K34, K35, and R36 between 80 and 100 ns (Figures 6I–K). The specificity of these interactions was validated by analyzing the MD trajectory of a TIRAP variant (K15 → A, K16 → A, K31 → A, and K32 → A). We observed that the PIP2 molecules were largely displaced from their relative positions (Figure 6G). However, the H-bond network involving K34, K35, R36, and PIP2 headgroups was conserved throughout the simulation (Figures 6H,I). Although we found that numerous DPPC molecules interact with basic and polar residues of the PBD, previous reports have concluded that only phospholipids are inefficient at anchoring TIRAP to the membrane. Moreover, absence or degradation of PIP2 by bacterial phosphatases markedly affected TIRAP’s membrane targeting ability (18).

### TIRAP Interacts with PIP3 in a PIP2-Analogous Manner

TIRAP has been previously observed to associate with the endosomal membrane, which contains PIP3 or lipids other than PIP2 (16, 17). To check the specificity of TIRAP for PIP3, we carried out a 100 ns MD simulation using a DPPC–PIP3 bilayer. The PIP2 molecules were replaced with PIP3 by manual superimposition, while other parameters were unchanged (Figure 7A). The TIRAP dimer had a stable backbone deviation (Figure 7B) in the presence of PIP3, with reasonable residue fluctuations (Figure 7C) and an overall compact tertiary fold, as indicated by a smooth decrease in the Rg values (Figure 7D). The order parameters of the acyl chains (−*S*_CD_ = 0.20) at carbons 15–31 of sn-2 and 34–50 of sn-1 chains were highly correlated with those of DPPC–PIP2 (Figure 7F). The average APL in the top and bottom leaflets were 59.47 and 61.17 Å^2^, respectively, which agrees with those of DPPC–PIP2. Calculation of headgroup density indicated that PIP2 and PIP3-containing bilayers have a similar profile, whereas the pure DPPC bilayer had a comparatively lower density (black dots) at both the top and bottom leaflets, indicating a relatively less dense membrane (Figure 7G). During the 100 ns dynamics trajectory, we observed that K15 and K16 of both monomers were consistently anchored to PIP3 by means of electrostatic interaction (Figure 7E). Meanwhile, PIP3 near K31 and K32 of the green monomer was displaced toward a positively charged cluster formed by K31 and K32 of the blue monomer and K34, K35, and R36 of both subunits. K32 and K35 formed consistent H-bonds with PIP3 headgroups, while R36 transitioned from electrostatic to H-bond interactions after 80 ns MD simulation (Figures 7H,I). The binding affinity indicated that TIRAP and its various segments have a comparatively stronger affinity toward PIP3 than PIP2 (Table 4). The total BFE of the TIRAP–PIP3 complex was estimated to be −4,709.26 kJ mol^−1^, while that of TIRAP–DPPC–PIP3 was −361.73 kJ mol^−1^, both of which are higher than those of TIRAP–PIP2 and TIRAP–DPPC–PIP2 complexes, respectively. Similarly, the PBD, the PBM, and their monomers all display a stronger affinity toward PIP3 than PIP2. Similar to the TIRAP–DPPC–PIP2 system, here also the PBD seems to play a greater role in PIP3 anchoring than PBM and whole TIRAP. Altogether, it appears that TIRAP has a higher specificity for PIP3 and can stably anchor to it on the cytoplasmic face of the endosomal membrane under physiological conditions.

**Table 4:** Binding free energy (kJ mol^−1^) between PIP3 and important segments of TIRAP.

Complex groups	Δ_vdW_^a^	Δ_elec_^b^	Δ_ps_^c^	Δ_SASA_^d^	Δ*G*_Total_^e^
TIRAP–PIP3	−65.09 ± 12.99	−6,794.62 ± 164.09	2,170.13 ± 140.45	−19.68 ± 4.10	−4,709.26 ± 128.97
TIRAP–DPPC–PIP3	−1,083.19 ± 59.30	−10,567.15 ± 423.84	12,114.78 ± 745.57	−826.17 ± 15.92	−361.73 ± 63.93
PBD–PIP3	−48.93 ± 12.32	−17,670.77 ± 200.37	2,200.74 ± 153.47	−17.27 ± 2.79	−15,536.22 ± 147.35
PBD–DPPC–PIP3	−404.50 ± 49.26	−19,439.01 ± 350.34	9,413.67 ± 545.66	−767.14 ± 15.64	−11,196.98 ± 689.82
PBM–PIP3	−13.45 ± 18.84	−12,776.36 ± 175.05	1,550.86 ± 160.01	−10.01 ± 2.16	−11,248.97 ± 96.74
PBM–DPPC–PIP3	−262.52 ± 50.35	−14,841.19 ± 280.99	7,811.93 ± 579.47	−752.95 ± 15.36	−8,044.73 ± 652.87
PBD_A–PIP3	−12.13 ± 18.88	−5,774.51 ± 138.30	1,267.30 ± 150.76	−9.79 ± 1.90	−4,529.13 ± 75.34
PBD_B–PIP3	−35.04 ± 14.05	−5,256.407 ± 165.15	957.26 ± 139.08	−8.23 ± 2.02	−4,342.42 ± 66.08
PBM_A–PIP3	−2.99 ± 14.37	−4,259.97 ± 101.86	984.71 ± 118.28	−5.58 ± 1.76	−3,283.82 ± 58.42
PBM_B–PIP3	−9.32 ± 12.57	−3,709.77 ± 138.89	656.25 ± 147.81	−5.15 ± 1.63	−3,068.01 ± 45.67

*^a^Van der Waals energy*.

*^b^Electrostatic energy*.

*^c^Polar solvation energy*.

*^d^Solvent accessible surface area energy*.

*^e^Total binding free energy*.

*DPCC, dipalmitoylphosphatidylcholine; PI, phosphatidylinositol; PBD, PI-binding domain; PBM, PI-binding motif; PIP3, PI (3,4,5)-trisphosphate; TIRAP, toll/interleukin 1 receptor domain-containing adaptor protein*.

## Discussion

TLR signaling pathways contain several TIR domain-containing adaptors, each with their own distinct function. While most TLRs employ MyD88-dependent NF-κB activation, TLR3 exclusively and TLR4 frequently employ TRIF-dependent IRF3 activation. Intriguingly, TLR2, TLR4, TLR7, and TLR9 pathways require an intermediate PI-anchored adaptor, TIRAP, for the recruitment of MyD88 to the activated TIR domains. Here, we constructed a full-length structure of TIRAP and performed extensive, multiscale MD simulations in the presence of model PI-containing bilayers to understand the process of membrane association and receptor–adaptor interactions at the atomic level.

We observed that PI molecules (PIP2/PIP3) play a crucial role in holding TIRAP on the model DPPC membranes, in that the MyD88 recruitment surface was exposed to water. PIs are multifunctional phospholipids that have significant local concentration-dependent impact on a number of signaling pathways (43, 44). These molecules provide membrane-anchoring ability to proteins lacking a definite transmembrane domain. The PI headgroups extend vertically beyond the membrane phospholipids, which are more planar (Figures S3,S5 in Supplementary Material). PIs induce vertical displacement of nearby phospholipids forming a stable membrane microdomain that can act as a protein anchoring unit (45, 46). The greater negative charge on the PI headgroups compared to that of membrane phospholipids creates a confined acidic environment for the charged residues of anchoring proteins (47). The X-ray crystal structures of PI–protein complexes have revealed that a conserved pattern of lysine-, arginine-, and histidine-rich surfaces or cavities are essential for interaction with PI headgroups (48). The long, flexible side chains of lysine and arginine have an electrostatic advantage over others for PI anchoring. The extensive basic patch at a specific location on the NTD indicates that the PI molecules must be closely positioned on the membrane for tight binding. We assumed that as many as four PI molecules could efficiently interact with the four distinct lysine-containing regions of the PBM. These regions are placed at the opposite ends of each PBM and, in the dimeric condition, a cluster of four PI-binding units could be formed, as shown in our TIRAP–DPPC–PIP2 MD simulation (Figure 5C). Since TIRAP acts as a bridge between MyD88 and TLR, the adaptor and receptor binding surfaces should be exposed to the cytoplasm. A pure DPPC bilayer was unable to hold the protein in a stable and well-exposed MyD88-interacting orientation; however, the DPPC–PIP2/PIP3 bilayer provided a mechanically less fluidic platform for TIRAP–MyD88 interaction. The AB loop is a unique feature of TIRAP that simultaneously interacts with MyD88 and TLR4 (20, 21). We found that although the AB loop from one subunit was partially immersed in the bilayer surface, the AB loop of the other subunit clearly oriented itself away from the membrane surface where MyD88 could easily bind. This behavior of the AB loop was also observed in the DPPC–PIP3 simulation, where the AB loop of one subunit remained solvent-exposed throughout the simulation. This suggests that partial immersion of the AB loop in the membrane surface is required for the stability of TIRAP.

Accumulating evidence indicates that TLR2 and TLR4 are localized to PIP2-rich compartment of the cell membrane, while TLR7 and TLR9 are found at phosphatidylserine-, PI(3,5)P2-, or PIP3-rich compartments of the endosomal membrane (16, 18, 49, 50). This suggests that TLR5 and TLR8 trigger immune signaling from distinct membrane regions that are devoid of local PI. It remains unclear why TIRAP is required by specific TLRs, while the homologous MyD88 alone is sufficient to trigger the TLR5- and TLR8-mediated NF-κB activation cascades. Further research might clarify the functional importance of this promiscuous scaffolding protein. It would be interesting to find out if all MyD88-dependent pathways require the assistance of TIRAP.

TIRAP lacks a functional domain to associate with downstream molecules as opposed to MyD88, which contains a death domain (DD) for the recruitment of IRAK4 (51). Despite sharing TIR-domain homology (Figure S6 in Supplementary Material), TIRAP and MyD88 possess distinct domain arrangements. MyD88 contains an N-terminal DD and a C-terminal TIR, which interacts with the C-terminal TIR of TLR and TIRAP simultaneously. In contrast, TIRAP has an N-terminal PBD for plasma membrane association in place of a DD. MyD88 exhibits different subcellular localization and is usually suspended in the cytoplasm until TIRAP links it to the activated TLR at the membrane. We assume that the capacity of TIRAP to recruit MyD88 is dependent on its stronger affinity for a PI-containing membrane, which facilitates a local, charge-dependent clustering of TLR, TIRAP, and MyD88 for specific immune signaling. Based on our simulation data, we can summarize the mechanism of TIRAP-PI integration in three key points. First, the long, negatively charged PI headgroups initially attract and trap the positively charged, elongated lysine or arginine side chains (e.g., K15, K16, K31, and K32) present in the PBM. Second, this initial electrostatic interaction allows partial absorption of the α helical PBM into the polar region of membrane phospholipids. At this stage, tryptophan and histidine residues also assist in membrane absorption because of their aromatic-polar chemical nature. Third, now having a suitable platform, PBD employs additional charged residues (K34, K35, and R36) to strengthen the attachment. These processes involve the formation and breakage of several non-bonded interactions along with the rotational and translational movements of TIRAP and membrane phospholipids (Movie S1 in Supplementary Materials).

Interestingly, we observed that during membrane association, most of the TIR surfaces remained exposed to the cytoplasm, indicating that PI allows physiological orientation of the TIR domain for interaction with its signaling partners (52). Although TIRAP shows a prototypical tertiary fold of TIR domains, it has very low sequence identity (17–24%) with known TIR structures. The crystal structures of TIRAP–TIR revealed that the BB loop signature with its preceding βB segment have poor electron densities, and are disordered (20, 21, 53, 54). Presumably, a long flexible AB loop connects αA to αB. This unique structural feature of TIRAP could be important for its specific physiological function as a bridging adaptor. Using mutagenesis and GST pull-down assays, Valkov et al. (21) proposed that the solvent exposed residues D96, L165, and S180 are essential for MyD88 binding. However, Lin et al. (20) showed that the AB loop mediates direct binding of TIRAP to MyD88. We found that D96/S180 as well as the AB loop surfaces are solvent exposed in the presence of the membrane, while L165 is packed within the dimer interface (Figure S7 in Supplementary Material). Purified TIRAP-TIR behaves as a monomer in solution, but it forms a homodimer *in vivo*, and so all crystal or solution structures reported to date are in a monomeric condition. This suggests that the interaction with the membrane or other signaling counterparts brings stability to the protein. The dimeric state of TIRAP–TIR has two different models: symmetric and asymmetric. The symmetric dimer, with its N-termini facing toward the membrane, is considered the physiological state of TIRAP (20, 21). The structures of other TIR domains have revealed different homodimerization interfaces, explaining the specificity of TIR–TIR interactions. For example, the TLR10–TIR dimer has an interface involving the αC helix, the BB loop, and the DD loop of both partners (55). Similarly, computational and mutagenesis studies have revealed that TLR4–TIR has a homodimer interface formed by the αC helix and the BB loop (56, 57). TLR4–TIR is thought to have an alternate interface involving the BB loop of the one and the αE helix of the other subunit (58). While TLR2 utilizes the αB, αC, αD helices and CD and DD loops of one monomer to pack against the αB helix and the BB loop of the other (59), TLR6 has a homodimer interface containing the CD loop, DD loop, and αC helices (60). The TIRAP–TIR dimer is formed by αC′ and αD helices from both the monomers. These differential homodimerization interfaces in structurally conserved TIR folds expose distinct surfaces for recruiting specific signaling counterparts.

TIRAP is an important scaffolding protein in the TLR signaling pathways and is also a well-validated drug target for treating a number of autoimmune and inflammatory diseases (22, 23). The peptides derived from the surface-exposed segments of TIRAP had been utilized to design decoy peptides that inhibit MyD88 recruitment by TLR2 and TLR4. Specifically, the AB loop peptide is a strong inhibitor of TLR2/TLR4-mediated signaling (22), while the BB loop peptide is not (61). This indicates that the AB loop of TIRAP has a greater potential for binding to TLR2/TLR4, and thus is a physiologically important segment, as observed in our simulation. The PBD carries a significant segment, the PBM, that could serve as a decoy to prevent or dissociate TIRAP from the membrane in order to control dysregulated and overexpressed TLR2/TLR4/TLR7/TLR9-dependent autoimmune diseases. This decoy peptide derivative, tagged with a cell-penetrating peptide, might let TIRAP localize to the membrane but prevent MyD88 recruitment, resulting in a temporary blocking of aberrant innate immune responses. While the structural information of individual segments of TIRAP are available, a full-length model of TIRAP interacting with the PI in the membrane will greatly improve our knowledge of the membrane association and orientation of key surface regions with respect to the receptors/adaptors. A complete view of TIRAP anchored to membrane-PIs is required for a broader understanding of the MAL^TIR^ protofilament formation (62) and myddosome assembly (51).

In the present study, we conclude that the N-terminal PBM—a rigid α helical structure essential for membrane association of TIRAP—aligns parallel to its dimeric counterpart. The PBM is partially submerged in the upper leaflet of the bilayer, and the PI-binding residues, present within an extensive basic patch, are stretched toward membrane phospholipids as a consequence of electrostatic attraction. Four distinct lysine-rich positively charged surfaces interact with the negatively charged PI headgroups through high-affinity H-bonds. The PBD alone has extraordinary affinity for PI compared to other segments of TIRAP, suggesting that this domain is essential for PI recognition. A PI-concentrated local membrane region is responsible for holding TIRAP in a pro-MyD88 orientation. In addition, we provide a low-energy, full-length structural model of TIRAP with excellent stereochemical parameters, which could be used in further structural studies to gain insights into the TLR2/TLR4/TLR7/TLR9-mediated pathways (TIRAP.pdb in Supplementary Material).

## Author Contributions

MP conceptualized, designed, and performed experiments. MP and SC analyzed the results and wrote the manuscript.

## Conflict of Interest Statement

The authors declare that the research was conducted in the absence of any commercial or financial relationships that could be construed as a potential conflict of interest.

## Footnotes

^1^http://plasma-gate.weizmann.ac.il/Grace/.

^2^Available at: http://nmr.chem.uu.nl/~adrien/course/molmod/get_timestamp.py.

^3^https://pymolwiki.org/index.php/Modevectors.

^4^https://pymolwiki.org/index.php/APBS.

## Supplementary Material

Movie S1 A low resolution movie clip showing interaction between toll/interleukin 1 receptor domain-containing adaptor protein (TIRAP) and the phosphatidylinositol (PI) 4,5-bisphosphate (PIP2) molecules in a dipalmitoylphosphatidylcholine (DPPC) bilayer. TIRAP is shown as secondary-cartoon representation (helices are lime; and sheets are orange colored). PIP2 is modeled as salmon spheres and PI-binding residues (K15, K16, K31, and K32) are represented as blue spheres. Only the phosphate (P8) atoms of DPPC bilayer are shown as transparent mauve beads for clarity.

Presentation S1 TIRAP.pdb. A computational model of the three dimensional structure of toll/interleukin 1 receptor domain-containing adaptor protein in homodimeric condition.
