# Supplementary material for: Insight into Phosphatidylinositol-Dependent Membrane Localization of the Innate Immune Adaptor Protein Toll/Interleukin 1 Receptor Domain-Containing Adaptor Protein
Source: Front Immunol. 2018 Jan 29;9:75. doi: 10.3389/fimmu.2018.00075 (PMC5796906; doi:10.3389/fimmu.2018.00075)
Supplement: Supplementary file 3 [file Data_Sheet_1.PDF]

Supplementary Materials

Insight into Phosphatidylinositol-Dependent Membrane Localization of the Innate Immune Adaptor Protein Toll/Interleukin 1 Receptor Domain-Containing Adaptor Protein

Mahesh Chandra Patra and Sangdun Choi*

Department of Molecular Science and Technology, Ajou University, Suwon 16499, Korea

***Corresponding author**
Sangdun Choi, Professor
Department of Molecular Science and Technology
Ajou University, Suwon 16499, Korea
Phone: +82-31-219-2600
Fax: +82-31-219-1615
E-mail: sangdunchoi@ajou.ac.kr

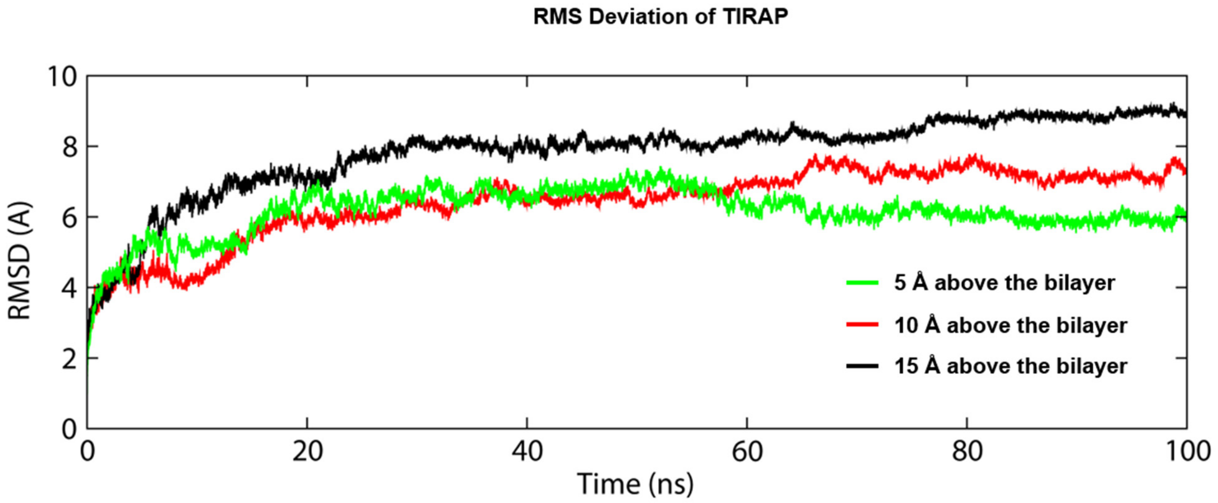

Figure S1. Backbone root mean square deviation (RMSD) of toll/interleukin 1 receptor domain-containing adaptor protein (TIRAP) in three different simulation systems. The RMSD of TIRAP at 5, 10, and 15 Å away from a dipalmitoylphosphatidylcholine–phosphatidylinositol 4,5-bisphosphate membrane surface are colored green, red, and black, respectively. The system where TIRAP was positioned 5 Å above the membrane maintained the most stable structure throughout the 100-ns-long molecular dynamics simulation.

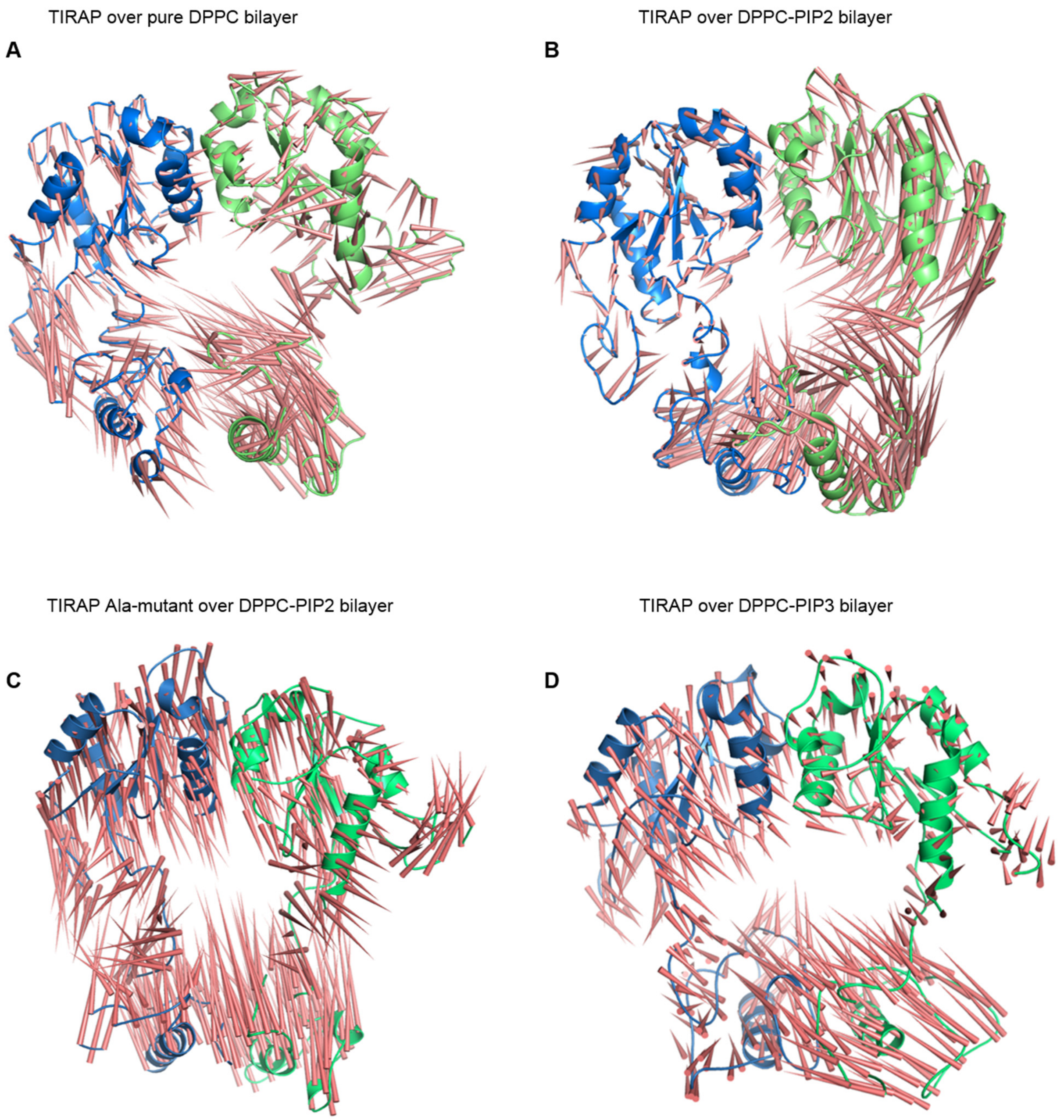

Figure S2. Porcupine plot representation of toll/interleukin 1 receptor domain-containing adaptor protein (TIRAP). (**A**) The global motion of TIRAP without phosphatidylinositol (PI) 4,5-bisphosphate (PIP2) on a dipalmitoylphosphatidylcholine bilayer. (**B**) The movement of TIRAP with PIP2 in the bilayer. (**C**) The movement of mutant TIRAP. (**D**) The movement of TIRAP with PI (3,4,5) trisphosphate in the bilayer. The plots show structural dynamics associated with the first eigenvector of each trajectory. The length and direction of spikes indicate the amplitude of motion during the molecular dynamics simulation. Each subunit is colored differently for ease of distinction.

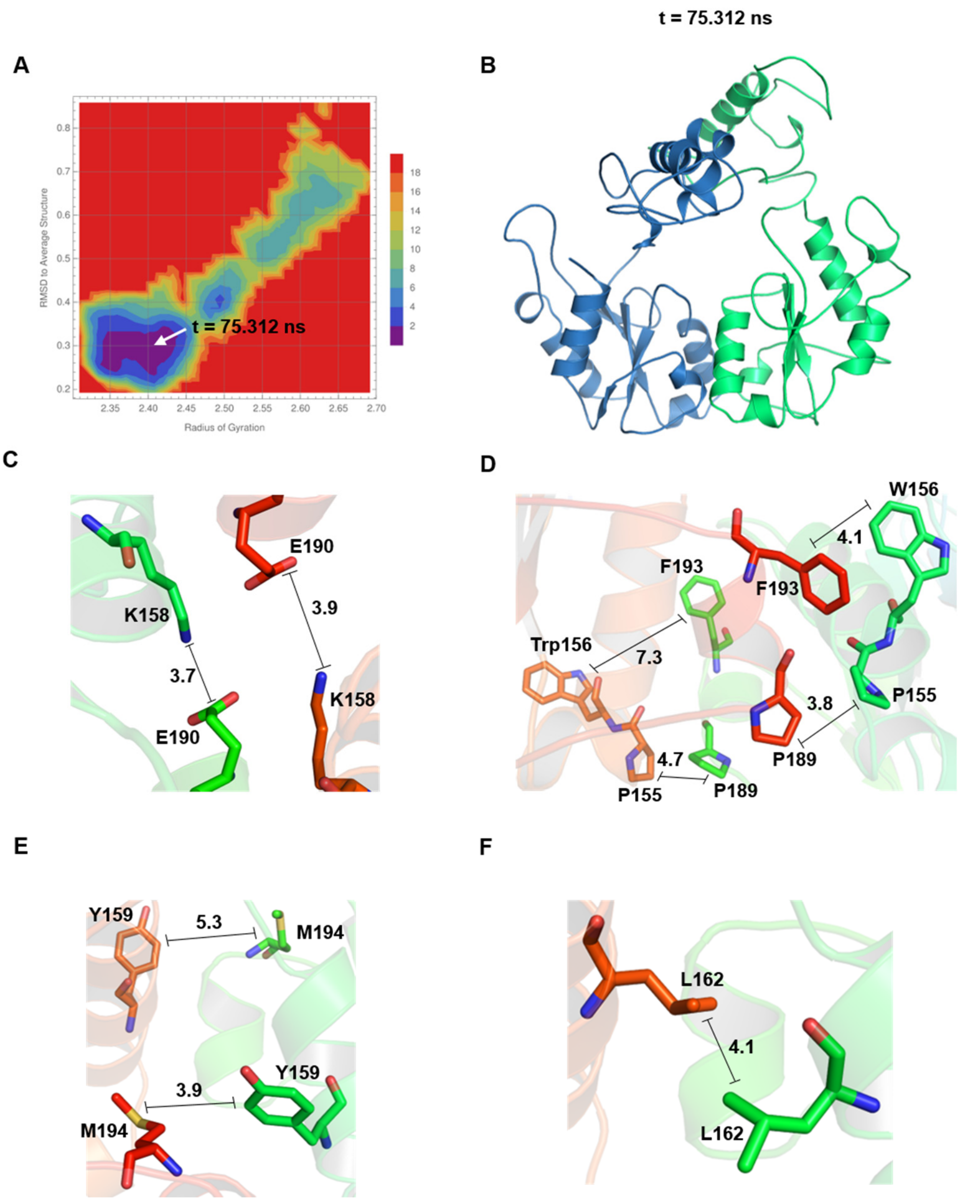

Figure S3. Accuracy check of modeled toll/interleukin 1 receptor (TIR) domain-containing adaptor protein (TIRAP) according to the known parameters. (**A**) Gibbs free energy landscape (FEL) showing different energetic states of TIRAP during 100 ns molecular dynamics simulation over a dipalmitoylphosphatidylcholine–phosphatidylinositol 4,5-bisphosphate bilayer. (**B**) A three-dimensional view of a representative low-energy conformation of TIRAP extracted from the 75,312th trajectory frame. This frame was chosen as it falls in the lowest energy region of the FEL as well as in the equilibrium plateau of root mean square deviation plots. (**C-F**) Dimer packing of residues belonging to the αC' and αD helices of both monomers of TIR domain.

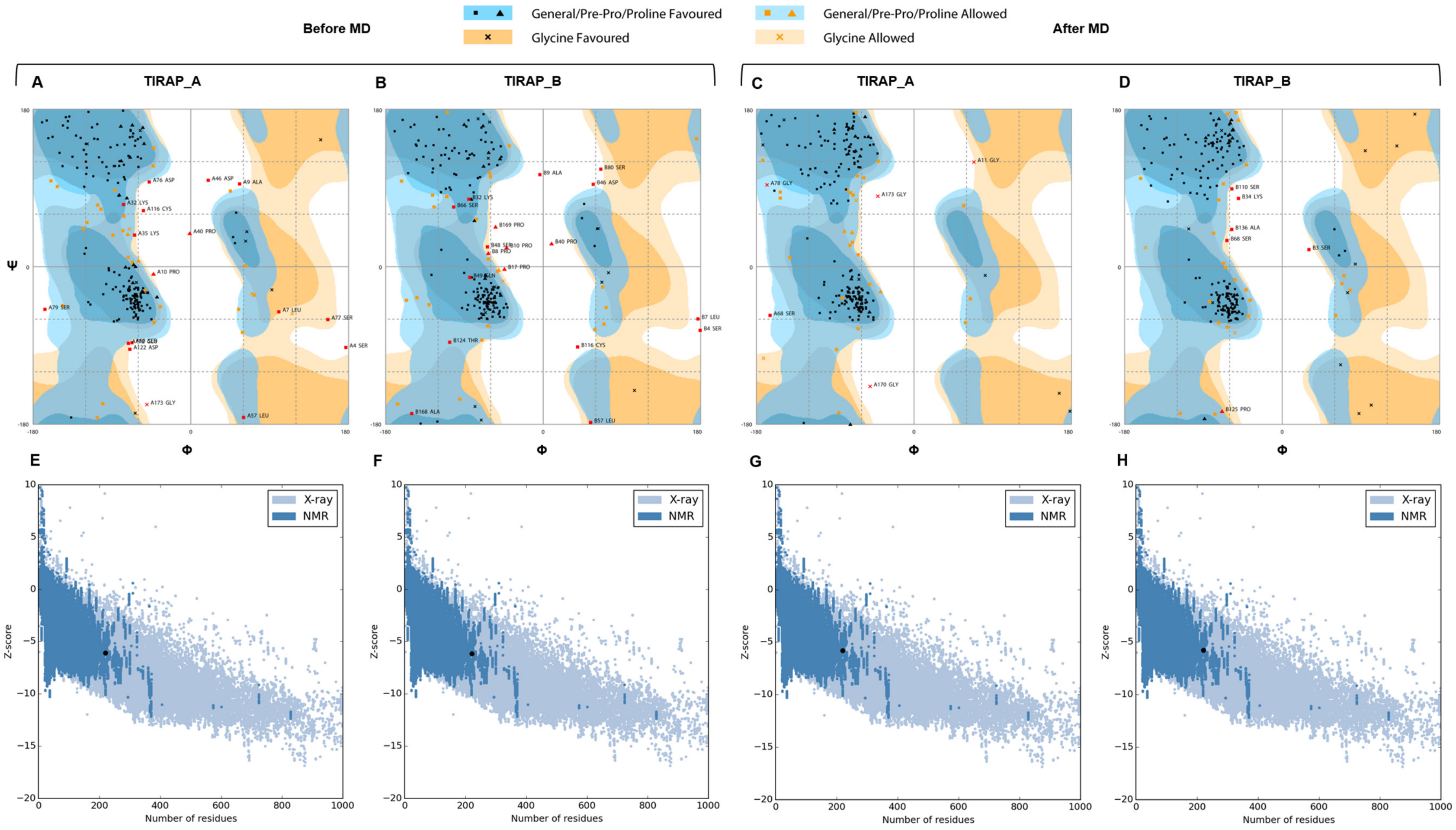

Figure S4. Model validation of toll/interleukin 1 receptor domain-containing adaptor protein (TIRAP) before and after molecular dynamics simulation. Top row (**A-D**) represent the Ramachandra plots of both chains of TIRAP and bottom row (**E-H**) represent ProSA-web Z-scores for both chains of TIRAP. X- and Y-axis of the Ramachandran plot indicates phi (Φ) and psi (Ψ) dihedral angles, respectively.

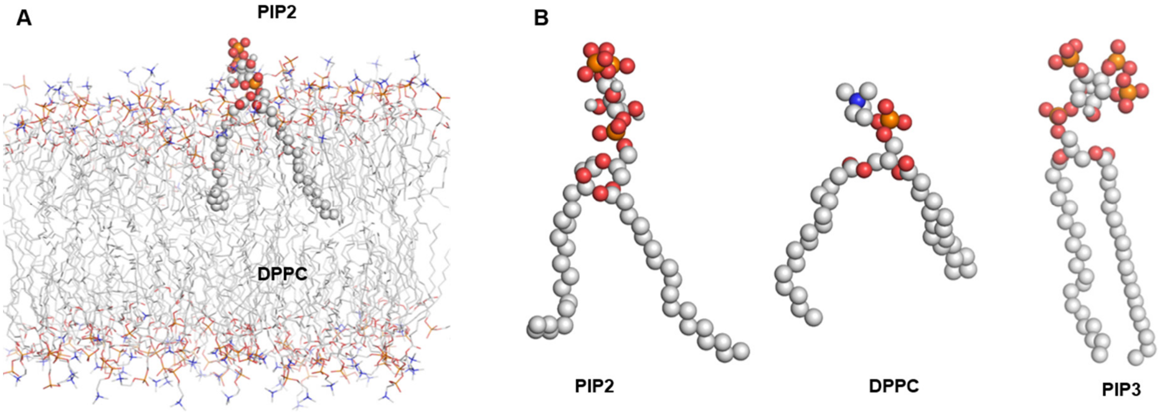

Figure S5. Size comparison of phosphatidylinositol (PI) 4,5-bisphosphate (PIP2), PI (3,4,5)-trisphosphate (PIP3), and dipalmitoylphosphatidylcholine (DPPC) molecules. (**A**) A DPPC bilayer of 128 phospholipids containing a single PIP2 molecule after a short 10 ns molecular dynamics (MD) simulation. (**B**) Alignment of a PIP2, a PIP3, and a DPPC molecule after a 10 ns MD simulation.

A

```
TIRAP_TIR-84-221     -------------------KDYD--VCVCHSEEDLVAAQDLVSYLEGSTASLRCFLQLRD
TLR4_TIR-653-839     KFYFHLMLLAGCIKYGRGENIYDAFVIYSSQDEDWVR-NELVKNLEEGVPPFQLCLHYRD
MyD88_TIR-159-296    -------------------ERFDAFICYCPSDIQFVQ--EMIRQLEQTNYRLKLCVSDRD
                                        : :*  :  . .: : *   :::  **     ::  :  **

TIRAP_TIR-84-221     ATPGGAIVSELCQALSS-SHCRVLLITPGFLQDPWCKYQMLQALTEAPGAEGCTIPLLS-
TLR4_TIR-653-839     FIPGVAIAANIIHEGFHKSRKVIVVVSQHFIQSRWCIFEYEIAQTWQFLSSRAGIIFIVL
MyD88_TIR-159-296    VLPGTCVWSIASELIEKRCRRMVVVVSDDYLQSKECDFQTKFALSLSPGAHQKRLIPIKY
                       ** .: :    .     .:  :::::  ::*.  * ::    * :    :    :  :

TIRAP_TIR-84-221     -GLSR---AAYPPELRFMYYVD------GR-GPDGGFRQVKEAVMRYL------------
TLR4_TIR-653-839     QKVEKTLLRQQVELYRLLSRNTYLEWEDSVLGRHIFWRRLRKALLDGKSWNPEGTVGTGC
MyD88_TIR-159-296    KAMKK----EFPSILRFITVCD---YT-NPCTKSWFWTRLAKALSLP-------------
                       :.:         *::          .       : :: :*:

TIRAP_TIR-84-221     ---QTLS-
TLR4_TIR-653-839     NWQEATSI
MyD88_TIR-159-296    --------
```

B

```
TIRAP_TIR-84-221     -------------------KDYDVCVC-HSEEDLVAAQDLVSYLEGSTASLRCFLQLRDA
TLR4_TIR-653-839     KFYFHLMLLAGCIKYGRGENIYDAFVIYSSQDEDWVRNELVKNLEEGVPPFQLCLHYRDF
                                        : **. *   *:::  . ::**. ** ..  ::  *: **

TIRAP_TIR-84-221     TPGGAIVSELCQ-ALSSSHCRVLLITPGFLQDPWCKYQMLQALTE--APGAEGCTIPLLS
TLR4_TIR-653-839     IPGVAIAANIIHEGFHKSRKVIVVVSQHFIQSRWCIFEYEIAQTWQFLSSRAGIIFIVLQ
                      ** **.::: : .: .*:  :::::  *:*. ** ::    * *      .  *  : :*.

TIRAP_TIR-84-221     GLSRAAYPPELRFM---------YYVDG-RGPDGGFRQVKEAVMRY--------------
TLR4_TIR-653-839     KVEKTLLRQQVELYRLLSRNTYLEWEDSVLGRHIFWRRLRKALLDGKSWNPEGTVGTGCN
                      :.::    ::.:            : *.  * .  :*::::*::

TIRAP_TIR-84-221     -LQTLS-
TLR4_TIR-653-839     WQEATSI
                       :: *
```

C

```
TIRAP_TIR-84-221     KDYDVCVCHSEEDLVAAQDLVSYLEGSTASLRCFLQLRDATPGGAIVSELCQALSSSHCR
MyD88_TIR-159-296    ERFDAFICYCPSDIQFVQEMIRQLEQTNYRLKLCVSDRDVLPGTCVWSI-ASELIEKRCR
                     : :*. :*:. .*:  .*:::  ** :.  *:  :. **. ** .: *  .. * ..:**

TIRAP_TIR-84-221     --VLLITPGFLQDPWCKYQMLQALTEAPGAEGCT-IPLLSGLSRAAYPPELRFMYYVDGR
MyD88_TIR-159-296    RMVVVVSDDYLQSKECDFQTKFALSLSPGAHQKRLIPIKYKAMKKEFPSILRFITVCDYT
                       *:::: .:**.  *.:*   **: :***.     **:      :  :*  ***:   *

TIRAP_TIR-84-221     GPDGGFRQVKEAVMRYLQTLS--
MyD88_TIR-159-296    NPCTKSWFW----TRLAKALSLP
                     .*            *   ::**
```

Figure S6. Sequence alignment of toll/interleukin 1 receptor (TIR) domains. (**A**) Alignment between TIR domains of Toll-like receptor 4 (TLR4), TIR domain-containing adaptor protein (TIRAP), and myeloid differentiation primary response 88 (MyD88). (**B**) Alignment between the TIR domains of TIRAP and TLR4. (**C**) Alignment between the TIR domains of TIRAP and MyD88.

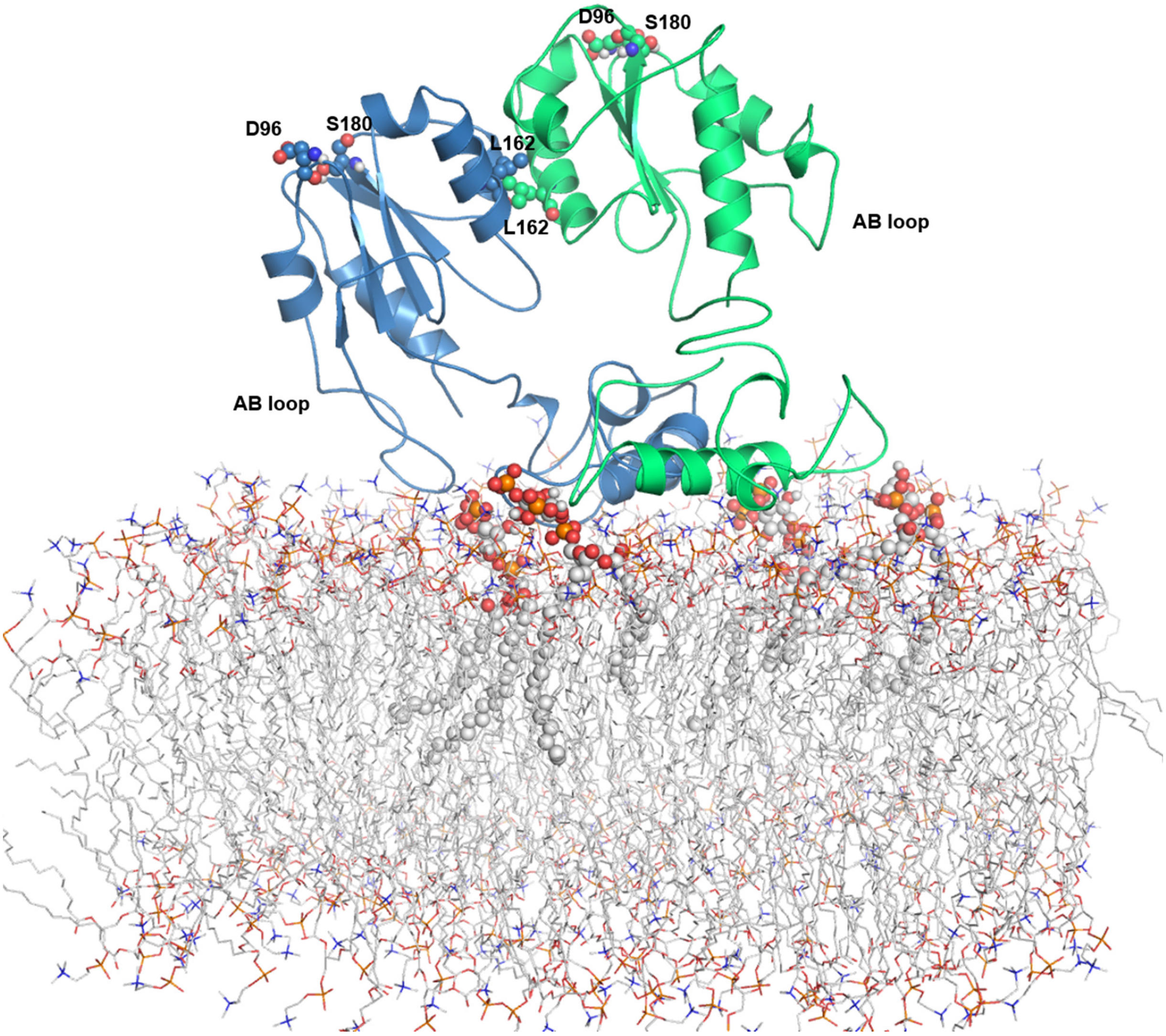

Figure S7. Potential myeloid differentiation primary response 88 (MyD88)-binding surfaces of toll/interleukin 1 receptor domain-containing adaptor protein. The residues highlighted as spheres and the AB loops are thought to interact with MyD88.

Table S1. Model validation scores of TIRAP based on Ramachandra plot and ProSA-web Z-scores.

Subunit	Ramachandran plot			ProSA-web Z score
**Before MD**	**Favored region**	**Allowed region**	**Outliers region**	
TIRAP_A	170 (78.3%)	30 (13.8%)	17 (7.8%)	-6.11
TIRAP_B	175 (80.3%)	25 (11.5%)	18 (8.3%)	-6.17
**After MD**				
TIRAP_A	185 (85.3%)	27 (12.4%)	5 (2.3%)	-5.8
TIRAP_B	187 (85.8%)	25 (11.5%)	6 (2.8%)	-5.79

TIRAP, toll/interleukin 1 receptor domain-containing adaptor protein; MD, molecular dynamics. % indicates total number of residues without glycine and proline.

Movie S1. A low resolution movie clip showing interaction between TIRAP and the PIP2 molecules in a DPPC bilayer. TIRAP is shown as secondary-cartoon representation (helices are lime; and sheets are orange colored). PIP2 is modeled as salmon spheres and PIP2 binding residues (K15, K16, K31, and K32) are represented as blue spheres. Only the phosphate (P8) atoms of DPPC bilayer are shown as transparent mauve beads for clarity.

Presentation 1. TIRAP.pdb. A computational model of the three dimensional structure of toll/interleukin 1 receptor domain-containing adaptor protein in homodimeric condition.
